# Multiomic Investigation of Shared Genetic Pathways in Paediatric Congenital Heart Disease and Neurodevelopmental Disorders

**DOI:** 10.1155/humu/7869246

**Published:** 2026-08-21

**Authors:** Jamie-Lee M. Thompson, Yunkai Gao, Eri Iwasawa, Debjani Das, Emma Rath, Michael Troup, David T. Humphreys, Haleh Heydarian, Julia Anixt, Nadine A. Kasparian, Tanya E. Froehlich, Jason Tchieu, K. Nicole Weaver, Congenital Heart Disease Synergy Study Group, Edwin P. Kirk, Russell Dale, Sally L. Dunwoodie, David S. Winlaw, Eleni Giannoulatou

**Affiliations:** ^1^ Victor Chang Cardiac Research Institute, Sydney, Australia, victorchang.edu.au; ^2^ School of Clinical Medicine, Faculty of Medicine and Health, University of New South Wales, Sydney, Australia, unsw.edu.au; ^3^ Cincinnati Children′s Hospital Medical Center, University of Cincinnati College of Medicine, Cincinnati, Ohio, USA, uc.edu; ^4^ Heart and Mind Wellbeing Center, Heart Institute and Division of Behavioral Medicine and Clinical Psychology, Cincinnati Children′s Hospital Medical Center, University of Cincinnati College of Medicine, Cincinnati, Ohio, USA, uc.edu; ^5^ NSW Health Pathology Randwick Genomics Laboratory, Sydney, Australia; ^6^ The Children′s Hospital at Westmead, University of Sydney, Sydney, Australia, sydney.edu.au; ^7^ Heart Center, Ann & Robert H. Lurie Children′s Hospital of Chicago, Chicago, Illinois, USA; ^8^ Feinberg School of Medicine, Northwestern University, Chicago, Illinois, USA, northwestern.edu

## Abstract

Recent medical advances have significantly improved the life expectancy of individuals with congenital heart disease (CHD); however, these children remain at increased risk of co‐occurring neurodevelopmental disorders (NDD), such as attention‐deficit/hyperactivity disorder and autism spectrum disorder. Although prenatal environmental factors, including placental dysfunction and altered oxygen levels in utero, as well as postnatal events such as cardiac surgery, may contribute to this increased risk, shared genetic factors may also underlie both conditions. Therefore, this study is aimed at investigating genomic, transcriptomic and epigenomic findings in patients with NDD and/or CHD using an integrative multiomic approach. A cohort of 14 trios and one duo was recruited: Two probands had both NDD and CHD, two had CHD only and 11 had NDD only. Blood samples were analysed using whole‐genome sequencing, RNA sequencing and DNA methylation profiling. We identified one large deletion (~2.5 Mb) and seven likely pathogenic/pathogenic (LP/P) variants, including two in autosomal dominant genes relevant to the patients′ phenotypes and five in autosomal recessive genes consistent with carrier status. This included a likely pathogenic de novo splice‐disrupting variant in the chromatin remodelling gene *ARID1B*, validated by RNA sequencing. DNA methylation analysis revealed epigenetic differences between CHD and NDD patients, with CHD patients showing elevated biological age acceleration. Comparison with a reference cohort of 178 controls identified four probands with extreme methylation dysregulation, including the individual with the *ARID1B* variant. Overall, these findings highlight the diagnostic and mechanistic value of integrative multiomic profiling in paediatric developmental disease cohorts.

## 1. Introduction

Neurodevelopmental disorders (NDD) are common comorbidities amongst patients with congenital heart disease (CHD), with an estimated prevalence of 35% of CHD patients reported to have some form of NDD, compared with 3%–11% in the general population [1, 2]. NDD includes a spectrum of conditions that alter cognitive, motor, emotional and behavioural development and may cause an increased risk of educational and behavioural problems. There are several known risk factors that contribute to the co‐occurrence of these conditions, including the severity of cardiovascular anomalies, need for neonatal cardiac surgery and prolonged hospitalisation and family socioeconomic disadvantage [3]. In utero, fetuses with severe CHD may experience reduced cerebral oxygenation and nutrient delivery due to abnormal cardiac physiology. These prenatal alterations in cerebral perfusion have been associated with impaired brain growth, including reductions in total and regional brain volumes [4]. Perioperative exposures in the neonatal period may impose additional physiological stress on the developing brain, further increasing vulnerability [5]. These established risk factors link CHD and NDD; yet, when combined, only explain up to 45% of the variability in neurodevelopmental outcomes. Therefore, it is hypothesised that other risk factors, such as genetics, may play a role.

Several recent studies have begun to investigate the genetic overlap between CHD and NDD and have uncovered variants in genes critical for early cardiac and neural development [6–9]. For example, CHD patients have an increased number of protein‐truncating and deleterious missense de novo variants in genes involved in neurogenesis, dendritic development and synaptogenesis [6]. Patients with CHD and NDD may also have greater numbers of protein‐damaging de novo mutations in genes highly expressed in the developing heart and brain compared with patients with isolated CHD [9]. Identification of these variants suggests there may be many developmental genes with a pleiotropic effect, contributing to the high prevalence of NDD within CHD patients; however, our understanding of the molecular pathways linking these disorders remains incomplete.

The aim of this study was to investigate shared molecular pathways between CHD and NDD by identifying genetic variants in developmentally relevant genes in probands with NDD and/or CHD. In addition, this study is aimed at using RNA sequencing and DNA methylation data to provide an overview of the gene expression and methylation changes associated with NDD and CHD.

## 2. Methods

A brief overview of methods is provided below; full details for all analyses are available in File S1.

### 2.1. Patient Recruitment and Ethics

Study participants were recruited at Cincinnati Children′s Hospital Medical Center, Ohio, United States. A total of 14 trios and one duo were enrolled, comprising probands with a confirmed diagnosis of NDD and/or CHD and their biological parents. Participants were consecutively enrolled without restriction by phenotype, severity or genetic aetiology, forming a heterogeneous, clinically referred pilot cohort. Written informed consent was obtained from all participants, including parental consent for minors. The study was approved by the Cincinnati Children′s Hospital Institutional Review Board (IRB ID 2021‐0493).

### 2.2. Sample Collection and Sequencing

Peripheral blood samples were collected from all participants. Genomic DNA and total RNA were extracted using standardised protocols. WGS was performed on 13 trios and one duo at ≥ 30x coverage using Illumina sequencing at the Broad Institute of MIT and Harvard. RNA sequencing was performed on a subset of nine trios and one duo (≥ 25 million paired‐end reads), and DNA methylation profiling was performed on 14 trios and one duo using the Illumina Infinium MethylationEPIC BeadChip array. Standard WGS quality control metrics (relatedness, sex confirmation, coverage, contamination and insert size) were assessed; all samples passed. The availability of whole‐genome sequencing, RNA sequencing and DNA methylation data for each participant is summarised in Table S1.

### 2.3. Annotation of Sequencing Data

WGS data were preprocessed and analysed according to the GATK Best Practices workflow [10]. Reads were aligned to GRCh38 using BWA‐MEM (v0.7.17) [11], variants called using GATK HaplotypeCaller with joint genotyping and quality filtered using variant quality score recalibration. Variants were annotated using ANNOVAR [12] and the Ensembl Variant Effect Predictor [13].

### 2.4. Variant Prioritisation

Variants with MAF < 1*%* in gnomAD (v2 and v3) [14] were assessed. Variants were considered potentially damaging if they altered protein length (nonsense, frameshift, in‐frame indel or splice site), were missense variants with REVEL ≥ 0.6 [15] or BayesDel ≥ 0.1 [16] or were exonic structural variants. Predicted‐damaging variants in NDD/CHD–associated genes (Table S2) were evaluated and verified by visual inspection using IGV [17]. For CHD trios, coding variants across all genes were additionally assessed under autosomal dominant (MAF < 0.001), autosomal recessive (MAF < 0.01), de novo and compound heterozygous inheritance models, excluding highly polymorphic genes. Variants of interest were classified according to ACMG guidelines. Genome‐wide high‐confidence LoF variants were identified using LOFTEE [14], filtered to no LoF flags, allele count < 20 in gnomAD 4.1, present in < 5 cases, read depth ≥ 8, allelic balance ≥ 0.15 and annotated to LoF‐intolerant genes (LOEUF ≤ 0.35).

### 2.5. Polygenic Risk Score Analysis

PRS were calculated for the nine probands of European ancestry using the pgsc_calc reproducible workflow [18], relative to a healthy elderly European control cohort (*n* = 3823) [19]. Scores were computed for ADHD (PGS003753) [20], anxiety (PGS001021) [21], autism (PGS000327) [22] and depression (PGS001829) [23]. PRS were transformed to control distribution percentiles; group differences were assessed using a Mann–Whitney *U* test with Benjamini–Hochberg correction.

### 2.6. RNA Sequencing Analysis

Reads were aligned to GRCh38 using STAR (v2.7.10) with two‐pass alignment. Aberrant expression was identified using OUTRIDER (v1.8.2) [24], supplemented with 97 RNA sequencing samples from children with complex medical disorders to improve outlier detection [25]. Six samples with size factors outside the 0.7–1.2 required range were excluded; the remaining 24 samples were analysed. Genes with adjusted *p* value < 0.05 were flagged as aberrantly expressed. Splicing variant validation and mono‐allelic expression (MAE) analysis were performed on the 10 trios with available transcriptomic data.

### 2.7. DNA Methylation Analysis

Methylation data were quality‐controlled using standard criteria; probes failing detection *p* value, bead count, cross‐reactivity, CpG‐SNP or sex chromosome filters were removed. One proband failed QC and was excluded. Beta values were normalised using functional normalisation. Epigenetic age was estimated using the Hannum blood‐specific and Horvath pan‐tissue clocks, which are optimised for blood samples and paediatric/adult cohorts, respectively; age acceleration was defined as the difference between predicted and chronological age. To account for family structure, linear mixed‐effects models were fitted with family as a random effect and age and sex as covariates; group differences were assessed using ANOVA with pairwise *t*‐tests.

### 2.8. Cross‐Cohort Differential DNA Methylation Analysis

To enable individual patient‐level methylation comparisons, the study cohort was integrated with two GEO reference control datasets: GSE74432 (*n* = 53, Illumina 450K [26] and GSE97362 (*n* = 125, Illumina 450K) [27]. Raw IDAT files were processed using minfi (v1.46.0) with preprocessNoob normalisation and dye bias correction. Batch effects were corrected using ComBat (sva v3.48.0) with negative control probe intensities as covariates; effectiveness was confirmed by PCA (Figure S1). Cell type composition was estimated using EpiDISH (v2.16.0) with the IDOL whole‐blood reference panel. A secondary epigenetic age analysis was performed using the EN and Levine clocks (selected for CpG coverage), with group differences assessed using ANOVA and Tukey′s HSD. For each proband, differentially methylated probes (DMPs) were identified using limma (v3.56.2) with design matrices incorporating group status, batch and cell type proportions, applying empirical Bayes moderation (FDR < 0.05). Probands with > 10,000 significant DMPs were classified as having methylation dysregulation.

### 2.9. Methylation Pathway Analysis

Within‐cohort differentially methylated regions (DMRs) were identified using the MethylMiner pipeline [28], (≥ 5 probes within a 500‐bp window). Pathway enrichment for genes annotated to proband‐specific DMRs was assessed using clusterProfiler [29] with Gene Ontology Biological Process terms [30, 31].

## 3. Results

### 3.1. Clinical Information

Detailed phenotypic and clinical information for all study participants is provided (Table 1). The cohort consisted of 10 male probands (66.7%) and 5 female probands. Four probands (26.7%) were diagnosed with hypoplastic left heart syndrome (HLHS), two of which were also diagnosed with a NDD condition. The most common form of NDD amongst the probands was ADHD, with 13 of 15 diagnosed. Other forms of NDD included autism spectrum disorder (ASD), learning disabilities, obsessive‐compulsive disorder (OCD) and oppositional defiant disorder. Eight probands had at least one parent with at least one diagnosed form of NDD, and of these probands, a further six had an extended family history of NDD.

**Table 1:** Phenotypic and clinical information for study participants.

Family	Individual ID	Relationship	Sex	Cardiac phenotype	Neurodevelopmental disorder	Psychiatric diagnosis	Genetically inferred ethnicity	Extended family history
1	1.001	Proband	M	HLHS	ADHD	Anxiety	AMR	No
1	1.002	Mother	F			Depression	AMR	No
1	1.003	Father	M			Depression	AMR	No
2	2.001	Proband	F	HLHS			AMR	No
2	2.002	Mother	F				AMR	No
2	2.003	Father	M				AMR	No
3	3.001	Proband	M		ADHD		EUR	Yes
3	3.002	Mother	F			Depression, PTSD	EUR	Yes
3	3.003	Father	M			Depression	EUR	Yes
4	4.001	Proband	M		ADHD, specific learning disability in reading	Anxiety	EUR	No
4	4.002	Mother	F		ADHD		AMR	No
4	4.003	Father	M		ADHD		EUR	No
5	5.001	Proband	F		ADHD, oppositional defiant disorder, dyslexia		EUR	No
5	5.002	Mother	F		ADHD	Anxiety	EUR	No
5	5.003	Father	M		ADHD	Anxiety	EUR	No
6	6.001	Proband	M		ADHD, intellectual disability		AFR	No
6	6.002	Mother	F		ADHD		AFR	No
6	6.003	Father	M		ADHD		AFR	No
8	8.001	Proband	M		ADHD, intellectual disability		AFR	Yes
8	8.002	Mother	F				EUR	Yes
9	9.001	Proband	M		ADHD, intellectual disability		EUR	No
9	9.002	Mother	F				EUR	No
9	9.003	Father	M		ADHD	Depression	EUR	No
11	11.001	Proband	M		ADHD		Unknown	No
11	11.002	Mother	F		ADHD, OCD	Anxiety	Unknown	No
11	11.003	Father	M				Unknown	No
12	12.001	Proband	F		ADHD, language disorder, learning disability		EUR	Yes
12	12.002	Mother	F				EUR	Yes
12	12.003	Father	M		Learning disabilities		EUR	No
13	13.001	Proband	M		ADHD, OCD, Tourette syndrome		AFR	No
13	13.002	Mother	F				EUR	No
13	13.003	Father	M		ADHD		AFR	No
15	15.001	Proband	M		ADHD, learning disability	Generalised anxiety disorder	EUR	Yes
15	15.002	Mother	F				EUR	Yes
15	15.003	Father	M				EUR	Yes
16	16.001	Proband	M	HLHS	ADHD	Anxiety	EUR	No
16	16.002	Mother	F			Anxiety	EUR	No
16	16.003	Father	M		ADHD		EUR	No
19	19.001	Proband	F		ADHD, ODD, nonverbal learning disorder		EUR	Yes
19	19.002	Mother	F				EUR	Yes
19	19.003	Father	M				EUR	No
20	20.001	Proband	F	HLHS		Major depressive disorder, Generalised anxiety disorder	EUR	Yes
20	20.002	Mother	F				EUR	Yes
20	20.003	Father	M				EUR	Yes

Abbreviations: ADHD, attention deficit/hyperactivity disorder; AFR, African; AMR, ad mixed American; EUR, European; HLHS, hypoplastic left heart syndrome; OCD, obsessive‐compulsive disorder; ODD, oppositional defiance disorder; PTSD, posttraumatic stress disorder.

### 3.2. WGS Variants

A total of 33 rare potentially deleterious variants and one large deletion were identified across the 14 probands included in this study, in genes known to be associated with either CHD and/or NDD (Tables 2, 3 and 4). We identified three pathogenic (P) and five likely pathogenic (LP) variants/deletions according to ACMG criteria. Of these, three variants/deletions were considered to have a likely impact on the probands′ phenotypes, occurring in autosomal dominant or de novo acting genes (Table 2), and five represented carrier findings in autosomal recessive disease genes (Table 3).

**Table 2:** Pathogenic/likely pathogenic variants considered disease‐causing in the proband.

Patient ID	Gene	Genomic (hg38)	Protein/transcript	Inheritance	gnomAD AF (v4.1)	ACMG
9.001	^∗^, *ALG3* ^∗^, *CLCN2* ^∗^, *CHRDL1* ^∗^, *ECE2*	NC_000003.12:g.183316907_185835082del		De novo	E = not found	P
					G = not found	
16.001	MYH6 ^∗^	NC_000014.9 : g.23396814A > C	NM_002470.4 : c.5658 + 3_5658 + 6del	Paternal	E = not found	LP
					G = not found	
19.001	ARID1B ^∗^^^^	NC_000006.12 : g.157198907G > A	NM_001374828.1 : c.4479G > A	De novo	E = fails QC	LP
					G = not found	

*Note:* The asterisk (∗) denotes gene identified in a Genomics England PanelApp panel related to either intellectual disability/autism or congenital heart disease (green, amber, or red evidence rating). (Genomics England PanelApp; https://panelapp.genomicsengland.co.uk, accessed October 2025). The caret symbol (^^^) denotes splice variant validated with RNAseq junction counts.

Abbreviations: ACMG, The American College of Medical Genetics and Genomics; AF, allele frequency; E, exome frequency; G, genome frequency; LP, likely pathogenic; P, pathogenic; VUS, variant of uncertain significance.

**Table 3:** Pathogenic/likely pathogenic variants in autosomal recessive genes (carrier status).

Patient ID	Gene	Genomic (hg38)	Protein/transcript	Inheritance	gnomAD AF (v4.1)	ACMG
2.001	*MYO7A* ^∗^	NC_000011.10 : g.77207295G > T	NP_000251.3:p.(E1917 ^∗^)	Maternal	E = 0.00000206	P
					G = not found	
3.001	*TWIST2* ^∗^	NC_000002.12 : g.238848457C > A	NP_001258822.1:p.(S81 ^∗^)	Paternal	E = 0	LP
					G = not found	
5.001	*GPR179* ^∗^	NC_000017.11 : g.38328525C > A	NP_001004334.3:p.(G1682 ^∗^)	Paternal	E = 0.0000103	LP
					G = 0.0000198	
15.001	*COL18A1* ^∗^^^^	NC_000021.9 : g.45509603 T > C	NM_001379500.1 : c.3495 + 2 T > C	Paternal	E = 0.00000151	LP
					G = not found	
20.001	*ACADM* ^∗^	NC_000001.11 : g.75732896 T > C	NP_000007.1:p.(M87T)	Maternal	E = not found	P
					G = not found	

*Note:* The asterisk (∗) denotes gene identified in a Genomics England PanelApp panel related to either intellectual disability/autism or congenital heart disease (green, amber, or red evidence rating). (Genomics England PanelApp; https://panelapp.genomicsengland.co.uk, accessed October 2025). The caret symbol (^^^) denotes splice variant validated with RNAseq junction counts.

Abbreviations: ACMG, The American College of Medical Genetics and Genomics; AF, allele frequency; E, exome frequency; G, genome frequency; LP, likely pathogenic; P, pathogenic; VUS, variant of uncertain significance.

**Table 4:** Variants of uncertain significance (VUS).

Patient ID	Gene	Genomic (hg38)	Protein/transcript	Inheritance	gnomAD AF (v4.1)	ACMG
1.001	*BCAR1*	NC_000016.10 : g.75229755 T > C	NP_055382.2:p.(H790R)	Paternal	E = 0.000000684	VUS
					G = not found	
1.001	*SETX* ^∗^	NC_000009.12 : g.132298174A > G	NP_055861.3:p.(L1896S)	Maternal	E = 0.00000616	VUS
					G = 0.00000657	
2.001	*PAX6*	NC_000011.10 : g.31801587C > A	NP_001355823.1:p.(V125F)	De novo	E = 0.00000137	VUS
					G = 0	
3.001	*DNMT3B* ^∗^	NC_000020.11 : g.32800234G > A	NP_008823.1:p.(G614E)	Maternal	E = 0.00000137	VUS
					G = 0.00000657	
4.001	*NEFH*	NC_000022.11:g.29490322_29490342del	NP_066554.2:p.(E895_K901del)	Maternal	E = not found	VUS
					G = not found	
5.001	*PANK2*	NC_000020.11 : g.3910812A > G	NP_001373322.1:p.(K296R)	Paternal	E = 0.0000951	VUS
					G = 0.000092	
5.001	*MYO7A* ^∗^	NC_000011.10 : g.77174781G > A	NP_000251.3:p.(R654H)	Paternal	E = 0.0000207	VUS
					G = 0.0000394	
5.001	*VPS13B* ^∗^	NC_000008.11 : g.99170112C > A	NP_689777.3:p.(P761H)	Maternal	E = 0.00019	VUS
					G = 0.0000987	
5.001	*GRM6* ^∗^	NC_000005.10 : g.178986615G > A	NP_000834.2:p.(R547C)	Maternal	E = 0.000271	VUS
					G = 0.000249	
6.001	*TYR* ^∗^	NC_000011.10 : g.89178215A > T	NP_000363.1:p.(T88S)	Paternal	E = 0.00000137	VUS
					G = 0.0000329	
6.001	*MARCHF8*	NC_000010.11:g.45464286_45464288del	NP_001269795.1:p.(S66del)	Maternal	E = 0.00000547	VUS
					G = 0.00000657	
12.001	*MYH3* ^∗^	NC_000017.11:g.10629838_10629841del	NC_000017.11(NM_002470.4): c.5658 + 3_5658 + 6del	Maternal	E = 0.000000684	VUS
					G = not found	
13.001	*NIPBL* ^∗^	NC_000005.10 : g.37049221C > T	NP_597677.2:p.(H2292Y)	Paternal	E = not found	VUS
					G = not found	
13.001	*CLCN7* ^∗^	NC_000016.10 : g.1448390C > T	NP_001278.1:p.(G660S)	Paternal	E = 0.0000123	VUS
					G = 0.0000394	
13.001	*TWIST1* ^∗^	NC_000007.14 : g.19116925 T > G	NP_000465.1:p.(K133Q)	Paternal	E = 0.000000684	VUS
					G = not found	
15.001	*PCDH19* ^∗^	NC_000023.11 : g.100296433A > C	NP_001171809.1:p.(Y1097 ^∗^)	Maternal	E = not found	VUS
					G = not found	
16.001	*RERE* ^∗^	NC_000001.11 : g.8355435G > A	NP_001036146.1:p.(Q1551 ^∗^)	Paternal	E = not found	VUS
					G = not found	
16.001	*OSGEP* ^∗^	NC_000014.9 : g.20449302C > A	NC_000014.9(NM_017807.4): c.412 − 36G > T	Paternal	E = fails QC	VUS
					G = not found	
19.001	*PLOD1* ^∗^	NC_000001.11 : g.11957873C > T	NP_000293.2:p.(P258L)	Paternal	E = 0.00000821	VUS
					G = 0.000046	
19.001	*CAD* ^∗^	NC_000002.12 : g.27243441G > A	NP_004332.2:p.(A2201T)	Paternal	E = 0.0000575	VUS
					G = 0.0000649	
20.001	*JAG1* ^∗^	NC_000020.11 : g.10672949C > A	NP_000205.1:p.(G47W)	Paternal	E = 0	VUS
					G = not found	
20.001	*AFG3L2* ^∗^	NC_000018.10 : g.12359961G > A	NP_006787.2:p.(R240W)	Paternal	E = 0.0000335	VUS
					G = 0.0000131	
20.001	*SLC46A1*	NC_000017.11 : g.28405167C > T	NP_542400.2:p.(R177H)	Paternal	E = 0.00000274	VUS
					G = 0.0000263	
20.001	*FOXN1* ^∗^	NC_000017.11 : g.28535093G > A	NP_001356298.1:p.(E508K)	Paternal	E = 0.0000351	VUS
					G = 0.0000263	
20.001	*CNTNAP2* ^∗^	NC_000007.14 : g.147486030C > A	NP_054860.1:p.(T589N)	Paternal	E = 0.0000089	VUS
					G = 0.0000131	
20.001	*FAM126A* ^∗^	NC_000007.14 : g.22961300C > G	NP_115970.2:p.(A256P)	Maternal	E = 0.000903	VUS
					G = 0.000671	

*Note:* The asterisk denotes genes identified in a Genomics England PanelApp panel related to either intellectual disability/autism or congenital heart disease (green, amber or red evidence rating) (Genomics England PanelApp: https://panelapp.genomicsengland.co.uk, accessed October 2025).

Abbreviations: ACMG, The American College of Medical Genetics and Genomics; AF, allele frequency; E, exome frequency; G, genome frequency; LP, likely pathogenic; P, pathogenic; VUS, variant of uncertain significance.

Amongst the variants with potential phenotypic relevance (Table 2), Proband 9 carried a de novo 2.5‐MB deletion on Chromosome 3 (NC_000003.12:g.183316907_185835082del), encompassing four developmental genes (*DVL3, ALG3, CLCN2, ECE2*). This deletion is likely to have contributed to the severe phenotype observed in Proband 9, who presented with ADHD, intellectual disability and microcephaly, consistent with haploinsufficiency of multiple neurodevelopmental genes. Of particular interest for CHD‐NDD overlap, Proband 16 (HLHS and ADHD) carried a paternally inherited stop‐gain variant in *MYH6* (NC_000014.9 : g.23396814A > C; NP_002462.2:p.Y724∗). In a large genomic study of 11,555 CHD probands, 4% of individuals with a *MYH6* variant carried an additional NDD diagnosis [32]; however, given that loss‐of‐function variants in *MYH6* are well‐tolerated in the general population (pLI = 0), this co‐occurrence likely reflects the broader CHD‐NDD comorbidity rather than a direct effect of *MYH6* on neurodevelopment. The same proband also carried a paternally inherited stop‐gain variant in *RERE.* This is a variant of uncertain significance, as it is located close to the end of the penultimate exon and is predicted to escape nonsense‐mediated decay, resulting in deletion of less than 1% of the protein. Pathogenic variants in *RERE* cause a syndromic form of intellectual disability in which ADHD and septal defects are recognised features [33]; it is therefore possible that this VUS may be relevant to this proband′s phenotype, although a contribution cannot be confirmed without further evidence. Finally, Proband 19 carried a de novo splice‐site variant in the chromatin remodelling gene *ARID1B* (NM_001374828.1 : c.4479G > A), an autosomal dominant gene in which de novo variants cause Coffin–Siris syndrome and intellectual disability [34]. Proband 19 had been diagnosed with ADHD and learning disabilities, consistent with this established disease mechanism.

Five further probands carried single heterozygous pathogenic or LP variants in autosomal recessive disease genes, representing carrier findings rather than direct disease‐causing genotypes (Table 3). Proband 2 with HLHS carried a maternal *MYO7A* stop‐gain variant (NC_000011.10 : g.77207295G > T; NP_000251.3:p.E1917∗), a gene associated with Usher syndrome type 1B and nonsyndromic deafness. ⁠Proband 3 carried a paternally inherited stop‐gain variant in *TWIST2* (NC_000002.12 : g.238848457C > A; NP_001258822.1:p.S81∗). *TWIST2* is associated with both autosomal dominant conditions (ablepharon‐macrostomia and Barber–Say syndromes), caused by missense variants and autosomal recessive Setleis syndrome, caused by biallelic loss‐of‐function variants [35]. As heterozygous loss‐of‐function variants in *TWIST2* are consistent with carrier status for Setleis syndrome rather than a dominant pathogenic mechanism, this variant was not considered causal for the proband′s phenotype.

Proband 5 carried a paternal *GPR179* stop‐gain variant (NC_000017.11 : g.38328525C > A; NP_001004334.3:p.G1682∗), primarily linked to congenital stationary night blindness, though emerging evidence suggests broader neurological involvement in some patients. Proband 15 carried a paternal *COL18A1* splice donor variant (NM_001379500.1 : c.3495 + 2 T > C; NC_000021.9 : g.45509603 T > C), associated with Knobloch syndrome, in which cognitive impairment and developmental delays are reported in the majority of cases. Proband 20 with HLHS carried a maternal pathogenic *ACADM* variant associated with medium‐chain acyl‐CoA dehydrogenase deficiency, a recessive condition which can present with developmental delays secondary to metabolic dysfunction [36]. These variants, found in autosomal recessive genes, indicate carrier status: A second pathogenic variant is required for disease manifestation. At present, all four genes are classified as AR; this may be re‐evaluated as new evidence emerges. Reporting these findings is important for genetic counselling and family planning.

The remaining 26 variants of uncertain significance (Table 4) included genes with strong biological plausibility, potentially warranting future functional studies.

Genome‐wide analysis of loss‐of‐function variants identified six variants in genes intolerant to loss of function (LOEUF ≤ 0.35) (Table 5). Two were previously identified (*PCDH19* and *RERE*), whereas four novel variants were found in *CHD9*, *CNTFR*, *INTS6* and *SPTBN1*. Three of these genes have established NDD associations. *CNTFR* variants have been linked to ADHD severity in multiple studies [37, 38], consistent with the ADHD phenotype in Proband 5. *SPTBN1* loss‐of‐function variants cause a spectrum from isolated ADHD to severe intellectual disability [39]; our patient presented with ADHD and intellectual disability, consistent with a potential pathogenic role. *INTS6* is a strong ASD candidate gene (SFARI database) and may have broader NDD associations, as suggested by the intellectual disability in Proband 8. The *CHD9* variant in Proband 6 lacks specific disease associations but warrants investigation given its chromatin remodelling function, consistent with other pathogenic variants in our cohort.

**Table 5:** List of putatively damaging variants identified in genome‐wide LoF analysis. Variants are annotated to a gene considered to be intolerant to loss of function (LOEUF ≤ 0.35).

Patient ID	Gene	Genomic (hg38)	Protein	Inheritance	gnomAD AF	LOEUF
5.001	*CNTFR*	NC_000009.12:g.34557589del	NP_671693.1:p.(I181 ^∗^)	Maternal	E = 0.00000274	0.339
					G = 0.00000657	
6.001	*SPTBN1*	NC_000002.12:g.54668402_54668403insCA	NP_003119.2:p.(K2310Tfs ^∗^113)	Maternal	E = not found	0.088
					G = not found	
6.001	*CHD9*	NC_000016.10:g.53209725_53209728del	NP_001295248.1:p.(K566Vfs ^∗^4)	Paternal	E = not found	0.17
					G = not found	
8.001	*INTS6*	NC_000013.11:g.51395410_51395411del	NP_036273.1:p.(F169Cfs ^∗^24)	De novo	E = 0.000000684	0.136
					G = not found	
15.001	*PCDH19* ^∗^	NC_000023.11 : g.100296433A > C	NP_001171809.1:p.(Y1097 ^∗^)	Maternal	E = not found	0.126
					G = not found	
16.001	*RERE* ^∗^	NC_000001.11 : g.8355435G > A	NP_001036146.1:p.(Q1551 ^∗^)	Paternal	E = not found	0.12
					G = not found	

*Note:* The asterisk means the identified by gene panel analysis.

Abbreviations: AF, allele frequency; E, exome frequency; G, genome frequency; LOEUF, loss of function observed/expected upper bound fraction score; LOF, loss of function.

### 3.3. Polygenic Risk Score Analysis

The cohort showed a trend towards higher PRS for ADHD and ASD compared with controls; however, due to the small sample size (*n* = 9), no score reached statistical significance (Figure S2). Despite this, individual‐level observations support a role for polygenic inheritance in shaping NDD risk, including in the context of identified rare variants.

Patient 4‐001 (NDD) had a PRS in the 89th percentile for ADHD and 83rd percentile for autism, with clinical diagnoses of ADHD and learning disability, and no LP/P monogenic variant identified. This represents the clearest case of a likely polygenic contribution to NDD phenotype in this cohort.

Patient 16‐001 (NDD + CHD) carries a paternally inherited *MYH6* truncating variant that is likely relevant to the cardiac phenotype. This patient also had an anxiety PRS in the 88th percentile and a clinical diagnosis of anxiety. Although the contribution of each genetic factor cannot be determined, this observation illustrates how common variant burden may coexist with rare variants in individuals with complex phenotypes.

Similarly, Patient 15‐001 (NDD) carries a paternally inherited LP variant in *COL18A1* (NM_001379500.1 : c.3495 + 2 T > C) in a gene associated with autosomal recessive disease, rendering the heterozygous finding unlikely to be fully explanatory. This patient scored in the 97th percentile for anxiety PRS with a corresponding anxiety diagnosis, again suggesting a polygenic contribution to the NDD phenotype.

Although formal epistasis testing is precluded by cohort size, these observations collectively suggest that polygenic burden may act additively with, or independently of, rare variant findings to shape neuropsychiatric outcomes in this cohort, a hypothesis warranting investigation in larger studies.

### 3.4. Gene Expression

There were 14 genes across three probands that were found to be aberrantly expressed (Table S3). Eight of these genes (*EIF4G1, EIF2B5, DVL3, AP2M1, ABCF3, SENP2, PSMD* and *PARL)* were downregulated in Proband 9, who was previously identified as having a large deletion on Chromosome 3, confirming the downstream effect of this finding. We investigated whether genetic variants could explain the other expression changes and identified that Proband 8 had one de novo variant of uncertain significance in *PPID* (NM_005038.3:c.737_738del), which may have contributed to aberrant *PPID* expression. No other variants of interest were identified in the remaining aberrantly expressed genes; however, these genes are involved in broad cellular processes that may influence pathways associated with NDD or CHD.

### 3.5. RNA Sequencing Validates Pathogenic Splicing Defects

Splicing variant validation was performed for three variants predicted to disrupt splice sites. Two showed definitive aberrant splicing. The *ARID1B* variant (NM_001374828.1 : c.4479G > A) in Proband 19 disrupts the splice donor consensus sequence, resulting in exon skipping absent in both parents, consistent with a de novo origin (Figure S3A). The paternally inherited *COL18A1* variant (NM_001379500.1 : c.3495 + 2 T > C) in Proband 15 disrupts the canonical splice donor site, producing two aberrant outcomes (cryptic donor activation with partial intron retention and full intron retention) both introducing a premature stop codon and predicting loss of function (Figure S3B). The *MYH3* variant (NM_002470.4 : c.5658 + 3_5658 + 6del) showed suggestive splicing changes, but junction coverage was below the threshold for confident validation (<20 reads). As detailed in the WGS analysis (Tables 2 and 3), variants in autosomal recessive genes (*COL18A1* and *MYH3*) are interpreted as carrier findings rather than disease‐causing in these probands.

### 3.6. MAE Analysis

Integration of DNA methylation with RNA sequencing data from 10 trios enabled assessment of MAE patterns. Of 5.6 million informative heterozygous SNPs with determinable parental origin, 223,753 (4.7%) had sufficient RNA coverage for allele‐specific expression analysis. The overall MAE rate was 0.3% (656 sites), within the expected normal range, with no family exceeding 1%, indicating no evidence of widespread allele‐specific silencing or imprinting defects.

### 3.7. Epigenetic Age Acceleration

**Figure 1:**
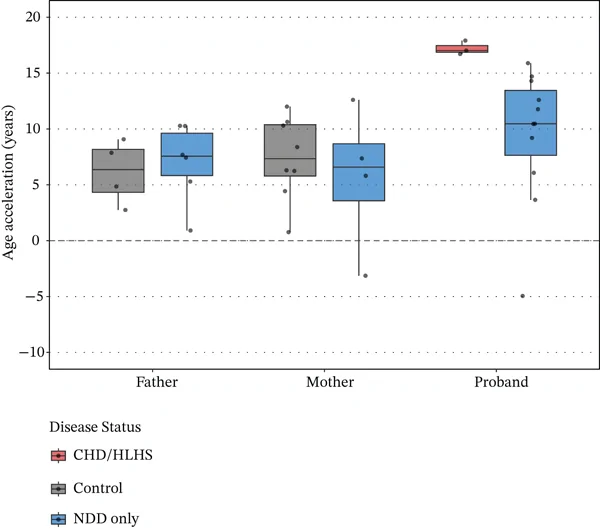
Epigenetic age acceleration in trios with CHD and/or NDD. Hannum age acceleration stratified by family relationship and disease status. Box plots showing the distribution of age acceleration (epigenetic age minus chronological age) calculated using the Hannum blood‐specific clock. The cardiac group (HLHS patients, *n* = 3) shows markedly higher age acceleration compared with control subjects (*n* = 12) and NDD‐only patients (*n* = 21). Each point represents an individual sample. Boxes indicate interquartile range (IQR), centre lines show medians and whiskers extend to 1.5 × IQR.

Analysis of epigenetic age revealed differences between patient groups (Figure 1). Both the Hannum and Horvath clocks showed a large biological age acceleration in HLHS patients compared with their chronological age. This acceleration was significantly greater than both the NDD‐only group (mean: 9.5 years greater, *p* = 0.006) and control subjects (mean: 7.4 years greater, *p* = 0.008), even after accounting for relatedness using mixed‐effects models. The NDD‐only probands showed moderate age acceleration that was not significantly different from controls (*p* = 0.31). Interestingly, when stratified by family role, probands across all disease categories showed higher age acceleration than their parents (probands: mean: 11.1 years; mothers: mean: 6.8 years; fathers: mean: 6.6 years).

A second analysis comparing the probands and healthy controls also revealed elevated biological aging in CHD probands (Figure S4). These alternate epigenetic clocks, EN and Levine, showed similar epigenetic age results for the CHD probands, but the EN clock had much higher ages for controls and NDD probands. Although both clocks confirmed a higher epigenetic age in CHD probands compared with NDD probands and controls, this result was not statistically significant.

### 3.8. Differential DNA Methylation Analysis

**Figure 2:**
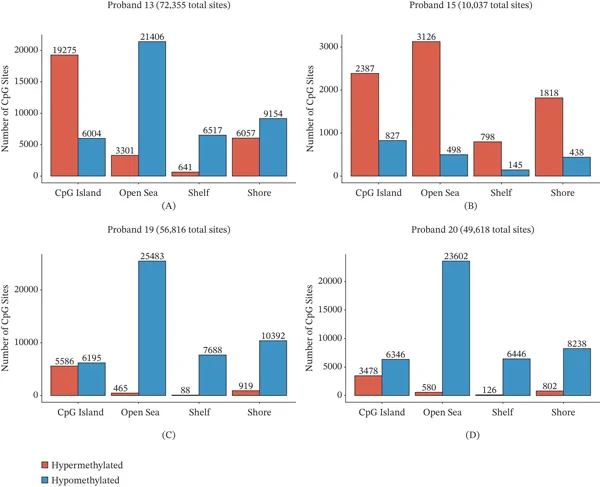
Differentially methylated positions in probands compared with controls. Individual proband methylation analysis identified four outlier cases with extreme methylation dysregulation (Probands 13, 15, 19 and 20) showing > 10,000 DMPs, whereas the remaining probands displayed moderate changes (149–6568 DMPs). Genomic context distribution shows distinct patterns across outlier probands: (A) Proband 13 (72,355 total DMPs) shows predominant hypomethylation in open sea regions (23,602 sites) with balanced hypermethylation in CpG islands (19,275 sites). (B) Proband 15 (10,037 DMPs) exhibits predominantly hypermethylation in CpG islands (2387 sites) and open sea regions (3126 sites). (C) Proband 19 (56,816 DMPs) displays extensive hypomethylation in open sea regions (25,483 sites) with hypermethylation in shores (10,392 sites). (D) Proband 20 (49,618 DMPs) shows a similar pattern with 23,602 hypomethylated open sea sites. DMPs were defined using limma comparison against 178 control samples (FDR < 0.05, |*Δ* *β*| > 0.2). Genomic contexts defined per Illumina MethylationEPIC array annotation. Definitions are as follows: CpG islands are regions with > 50% GC content, > 60% CpG observed/expected ratio and minimum length of 200 bp; shores are regions flanking CpG islands within 2 kb (2000 bp); shelves are regions 2–4 kb (2000–4000 bp) from CpG islands; and open sea regions are isolated CpGs located > 4 kb (4000 bp) from any CpG island.

Cross‐cohort differential methylation analysis comparing individual probands to controls (*n* = 178) revealed methylation dysregulation in four probands (Figure 2). Proband 13 showed the most severe dysregulation with 72,355 DMPs, followed by Proband 19 (56,816 DMPs), Proband 20 (49,618 DMPs) and Proband 15 (10,037 DMPs). All remaining probands had fewer than 10,000 DMPs.

The genomic distribution of DMPs varied substantially across probands (Figure 2). In Proband 13, hypermethylated sites were enriched in CpG islands (19,275 sites, 76.2% of CpG island DMPs) whereas hypomethylated sites were predominantly in open sea regions (21,406 sites, 86.6% of open sea DMPs), indicating disruption of normal methylation maintenance mechanisms rather than targeted changes at specific loci. Probands 19 and 20 had similar methylation patterns across genomic contexts, with greater levels of hypomethylation in CpG islands and markedly greater levels of hypomethylation across open seas, shelves and shores. Finally, Proband 15 had quite a different pattern of methylation dysregulation, showing higher levels of hypermethylation across all genomic contexts.

### 3.9. Pathway Enrichment Analysis of DNA Methylation Regions

DMRs were enriched for pathways related to both cardiac and neurological processes (Table S4). Key pathways included *muscle tissue development, muscle cell differentiation* and *striated muscle cell differentiation,* which align with the structural and functional roles of muscle tissues in cardiac development. Pathways such as *regulation of blood circulation* and *regulation of heart contraction* further highlight the involvement of these DMRs in cardiac‐specific biological processes. Additionally, enrichment in pathways related to neuronal development, such as *axon guidance, neuron projection guidance, synapse organisation* and *transmission of nerve impulse*, suggests a role for these epigenetic changes in nervous system function. Processes like *stem cell differentiation, cell fate commitment* and *pattern specification process* show broader developmental dysregulation.

## 4. Discussion

This study applied an integrative multiomic framework to a small cohort of paediatric NDD and CHD probands to characterise individual‐level genomic, transcriptomic and epigenetic findings. These analyses demonstrate how genomic, transcriptomic and epigenomic data can provide complementary diagnostic and mechanistic information at the individual‐patient level. These include rare variants in developmental and chromatin remodelling genes, heterogeneous methylation patterns across probands and epigenetic age estimates that, although variable and limited by small sample size, suggest that this framework may identify molecular subgroups within the broader NDD/CHD population. We interpret these findings as hypothesis‐generating, acknowledging that the small cohort size and overlapping confidence intervals across groups preclude definitive conclusions.

Whole genome sequencing identified 33 rare potentially deleterious variants and one microdeletion across 14 probands. The most significant finding was a de novo splice‐site variant in the chromatin remodelling gene *ARID1B* in Proband 19, functionally confirmed by RNA sequencing, which demonstrated aberrant splicing absent in both parents. This variant likely underlies the widespread methylation dysregulation observed in this proband. The SWI/SNF complex, of which *ARID1B* is a component, regulates chromatin accessibility during heart and brain development, and loss of function can produce genome‐wide epigenetic consequences [40, 41]. Chromatin‐disrupting variants have also been implicated in other neuropsychiatric conditions including OCD [42], supporting dysregulation of this machinery as a mechanism relevant across neurodevelopmental and psychiatric phenotypes.

The remaining probands with extreme methylation dysregulation showed distinct and heterogeneous patterns. Proband 13 (NDD), despite no confirmed causal variant, exhibited 72,355 differentially methylated sites with a spatially distinct signature of hypermethylation enriched at CpG islands and hypomethylation in open sea regions, suggesting disruption of methylation maintenance rather than global loss. Probands 19 and 20, despite having different clinical presentations (NDD and CHD, respectively), showed strikingly similar methylation profiles across genomic contexts, consistent with shared underlying genetic factors. In contrast, Proband 15 (NDD) showed enriched hypermethylation across all genomic contexts, suggesting global dysregulation of methylation machinery with potential for broad transcriptional repression in developmental networks.

Our results highlight both shared and distinct genetic factors contributing to NDD and CHD. Several genes identified in our cohort demonstrate pleiotropic effects across cardiac and neural tissues. Proband 16 has an *MYH6* variant which may be relevant to this individual′s cardiac phenotype (although most reported patients with pathogenic variants in this gene have had much less severe cardiac manifestations than HLHS). This individual also has an inherited truncating variant in *RERE*; this is a variant of uncertain significance but could possibly contribute to the neurodevelopmental phenotype. This observation aligns with recent large‐scale sequencing studies suggesting that patients with both CHD and NDD carry a greater burden of damaging variants compared with those with isolated conditions [9, 43].

The epigenetic analysis revealed a substantial biological age acceleration in HLHS patients, significantly greater than both NDD‐only patients (mean: 9.5 years greater) and controls (mean: 7.4 years greater). This magnitude exceeds previous reports in paediatric disease cohorts and approaches levels typically observed in severe adult‐onset conditions [44–47], suggesting CHD is associated with broader biological effects beyond the cardiovascular system, though replication in larger cohorts is needed. This acceleration may reflect chronic hypoxia and surgical stress, though formal investigation of these mechanisms is needed. Our group recently demonstrated that CHD patients carry significantly higher polygenic risk scores for hypertension, Type 2 diabetes and obesity compared with healthy controls [48], suggesting both genetic predisposition and accelerated biological aging may contribute to the increased comorbidity burden.

Several limitations should be considered when interpreting these findings. The small sample size (15 probands) limits statistical power for detecting associations and may not capture the full spectrum of genetic variation relevant to both conditions. The study was designed to investigate shared pathways across patients with NDD and/or CHD; however, only two probands had both diagnoses, which limits direct comparison between isolated and comorbid presentations. Future studies with larger coaffected cohorts would better enable such comparisons. The absence of a matched control group for genomic analyses limits interpretation of methylation patterns, although this was taken into consideration for the analyses performed in this study.

The heterogeneity of both cardiac and neurodevelopmental phenotypes within our cohort presents challenges for identifying convergent mechanisms. Although ADHD was the predominant NDD diagnosis, the inclusion of ASD, intellectual disability, and other conditions may obscure condition‐specific genetic factors. Future studies with larger, phenotypically stratified cohorts will be essential to validate and extend these findings. Additionally, functional validation of identified variants through cellular or animal models would strengthen causal inferences, particularly for variants of uncertain significance.

Our integrated multiomic approach identified molecular findings relevant to both CHD and NDD, including variants affecting chromatin remodelling and heterogeneous patterns of epigenetic dysregulation. These observations support further investigation of shared developmental mechanisms in larger cohorts. The pathway enrichment analysis, which identified involvement of both muscle development and neuronal guidance, suggests possible molecular links between cardiac and neural development. This is in line with recent single‐cell transcriptomic studies indicating some shared progenitor populations during early embryogenesis [49]. Collectively, these findings highlight the value of integrative genomic and epigenomic profiling for capturing the complexity of these disorders and improving diagnostic precision for affected children. Larger, phenotypically stratified cohorts and functional validation studies will be necessary to clarify causality and determine the full clinical implications of these observations.

## Author Contributions

J.M.T. led the multiomics analysis, performed data analysis and statistical analyses, produced all figures and wrote the manuscript. Y.G. developed and executed data processing pipelines and contributed to variant curation. E.I. was responsible for patient recruitment, sample collection and generation of multiomic datasets at Cincinnati Children′s Hospital. D.D. and E.R. contributed to variant curation. M.T. developed and executed genomic data processing pipelines. D.H. designed and implemented transcriptomic analysis pipelines. H.H., J.A., N.A.K., T.E.F., J.T. and K.N.W. contributed to clinical data collection, patient phenotyping and neurodevelopmental outcome assessment. R.D. provided scientific expertise in neurodevelopmental disorders and contributed to data interpretation. The Congenital Heart Disease Synergy Study Group contributed to funding acquisition and the development of analytical pipelines used in this study. E.P.K. provided scientific expertise and contributed to data interpretation. S.L.D., D.S.W. and E.G. conceived and supervised the study and acquired funding.

## Funding

This study was supported by the National Health and Medical Research Council (10.13039/501100000925) (1181325, 1135886, 2007896, 2018360). Open access publishing was facilitated by the University of New South Wales, as part of the Wiley‐University of New South Wales agreement via the Council of Australasian University Librarians.

## Disclosure

All authors reviewed and approved the final manuscript.

## Conflicts of Interest

The authors declare no conflicts of interest.

## Supporting information


**Supporting Information** Additional supporting information can be found online in the Supporting Information section. File S1: Full description of patient recruitment, sample collection, sequencing, variant prioritisation, polygenic risk score analysis, RNA sequencing, mono‐allelic expression analysis, DNA methylation analysis, cross‐cohort differential methylation analysis and methylation pathway analysis. Figure S1: Assessment of batch correction for cross‐cohort DNA methylation data using principal component analysis (PCA). Figure S2: Polygenic risk scores for common neurodevelopmental and psychiatric phenotypes in patients with NDD and/or CHD compared with healthy control subjects. Figure S3: RNA sequencing validation of splice‐site variants. Figure S4: Epigenetic age estimates by disease group using EN and Levine clocks. Table S1: Data types available for each study participant. Table S2: Neurodevelopmental disorder (NDD) and congenital heart disease (CHD) genes used for variant curation. Table S3: Genes with aberrant expression identified by RNA sequencing. Table S4: Pathway enrichment analysis of differentially methylated regions.

## Data Availability

The data that support the findings of this study are available on request from the corresponding author. The data are not publicly available due to privacy or ethical restrictions. Code used in this analysis has been made available on GitHub (https://github.com/VCCRI/NDD_multiomic_study_2025).
