# Supplementary material for: Multiomic Investigation of Shared Genetic Pathways in Paediatric Congenital Heart Disease and Neurodevelopmental Disorders
Source: Hum Mutat. 2026 Aug 21;2026:7869246. doi: 10.1155/humu/7869246 (PMC13498852; doi:10.1155/humu/7869246)
Supplement: Supplementary file 1 [file HUMU-2026-7869246-s001.docx]

**Supplementary Material**

**Multi-omic investigation of shared genetic pathways in paediatric**

**congenital heart disease and neurodevelopmental disorders**

Thomson JM *et al*

**Supplementary Methods**

*The text below provides the full methods detail. The main manuscript contains a condensed methods section; this supplementary version includes additional detail on quality control, software versions, and analytical parameters omitted from the main text.*

**Patient recruitment and ethics**

Study participants were recruited at Cincinnati Children’s Hospital Medical Center in Ohio, USA. A total of 14 trios and one duo were recruited, which included probands diagnosed with NDD and/or CHD, along with their biological parents. Inclusion criteria included a confirmed clinical diagnosis of NDD and/or CHD. This was a heterogeneous, clinically referred pilot cohort; participants were enrolled consecutively as they presented to the clinic with a confirmed diagnosis, without further restriction by phenotype, severity, or genetic aetiology. Written informed consent was obtained from all participants, including parental consent for minors. The study was approved by the Cincinnati Children’s Hospital Institutional Review Board (IRB ID 2021-0493).

**Sample collection and sequencing**

Peripheral blood samples were collected from all 15 probands and their parents. Genomic DNA and total RNA were extracted using standardised protocols (availability of data types detailed in Supplementary Table 1). Whole-genome sequencing (WGS) was performed on samples from 13 trios and one duo at a minimum coverage of 30x using Illumina sequencing. Quality control processes were carried out to check for relatedness, sex confirmation, coverage, contamination, insert size, and jumping metric (3-5). All samples passed for all metrics and proceeded to downstream analysis. For transcriptomic analysis, RNA sequencing (RNAseq) was performed on a subset of nine trios and one duo, with a read depth of at least 25 million paired-end reads. Epigenetic profiling for 14 trios and one duo was performed using the Illumina Infinium MethylationEPIC BeadChip array. All sequencing and arrays were performed by The Broad Institute of MIT and Harvard, Cambridge, Massachusetts.

**Annotation of sequencing data**

WGS data were pre-processed and analysed according to the Genome Analysis Toolkit (GATK) Best Practices workflow (6). Sequencing reads were aligned to the GRCh38 human reference genome using BWA-MEM (v0.7.17) (7). Variants were called using GATK’s HaplotypeCaller in genomic VCF mode for each sample, followed by joint genotyping across all samples with GATK GenotypeGVCFs. Variant quality score recalibration was used to filter out low-quality single nucleotide variants. Variants were annotated using ANNOVAR (8) and the Ensembl Variant Effect Predictor (9).

**Variant prioritisation**

Variants with a minor allele frequency (MAF) less than 1% in the Genome Aggregation Database (gnomAD v2 and v3) (10) were assessed. Variants were considered potentially damaging if they – a) altered the protein length, such as nonsense, frameshift, in-frame insertion/deletion and splice site variants, or b) were missense variants with a REVEL (11) score ≥0.6 or a BayesDel (12) score ≥0.1, or c) were structural variants in exonic regions. Predicted-damaging variants in genes known to be associated with NDD and/or CHD (Supplementary Table 2) were evaluated. Variants of interest were verified by visual inspection of the variant locus using the Integrative Genomics Viewer (IGV) (13). For the CHD trios, predicted-damaging variants in all coding genes were also evaluated, excluding highly polymorphic genes susceptible to high false-positive variant calls, such as mucins. IGV-verified coding variants were curated and assessed under autosomal dominant (AD), autosomal recessive (AR), de novo, and compound heterozygous inheritance models. While an initial MAF threshold of <1% was applied, more stringent thresholds were used for inheritance-specific analyses (MAF <0.001 for AD and <0.01 for AR). VarSome was used to support ACMG classification of each variant of interest (14).

Loss-of-function variants across the whole genome were identified using LOFTEE (10). Variants were filtered by those that met the following criteria: high confidence LoF variants with no flags, allele count < 20 in gnomAD 4.1, present in fewer than 5 cases, read depth ≥ 8, allelic balance ≥ 0.15, and annotated to a gene considered to be intolerant to loss of function (LOEUF ≤ 0.35).

**Polygenic risk score analysis**

A healthy elderly European control cohort (3,823 individuals) was used for comparison (15). Only the nine probands with European ancestry were used for this sub analysis (n=1 CHD only, n=1 CHD + NDD, n=7 NDD only). Polygenic scores for each participant were calculated with the *pgsc_calc* reproducible workflow (16-21). PRS were calculated for common NDD and psychiatric conditions including ADHD (Polygenic Score Catalog ID: PGS003753; 35,445 variants) (22), anxiety (PGS001021; 9,747 variants) (23), autism (PGS000327; 35,087 variants) (24), and depression (PGS001829; 7,534 variants) (25). PRS were transformed into percentiles of the control PRS distribution and differences in percentile distributions were compared using a Mann-Whitney U test. Benjamini and Hochberg correction for multiple testing was separately applied to *P* values for each cohort.

**RNA sequencing analysis**

RNA sequencing (RNAseq) data underwent quality control using FastQC (v0.12.0). All samples passed quality metrics with per-base sequence quality scores >30, no adapter contamination detected, and sequence duplication levels within expected ranges for RNA-seq libraries. No samples were excluded based on QC metrics. High-quality reads were aligned to the GRCh38 human reference genome using STAR (v2.7.10) (26) with default two-pass alignment to improve splice junction detection.

We identified genes with aberrant expression by analysing gene-level count data in OUTRIDER (v1.8.2) (27). To enable robust detection of expression outliers, an additional 97 RNA sequencing samples from children with complex medical disorders were included to estimate the background expression distribution and improve outlier detection (28). OUTRIDER uses a statistical modelling approach to account for shared sources of variation across samples before identifying genes with aberrant expression. This approach implicitly accounts for confounding effects, including potential cohort-related differences, without requiring explicit batch covariates. Six samples had a size factor outside the 0.7-1.2 range required for OUTRIDER to work (1.002, 2.001, 2.003, 3.002, 20.001, 20.002), so were excluded and the remaining 24 samples used for analysis. Genes exhibiting significant deviations (adjusted p-value < 0.05) were flagged as candidates for aberrant expression.

**Splicing variant validation**

To validate predicted splicing variants, junction-level analysis was performed on RNA sequencing data from 10 trios with available transcriptomic data. Splice junctions were extracted from STAR-aligned BAM files. For each variant predicted to affect splicing, we examined junctions within a 1000bp window around the variant site. Aberrant splicing was confirmed when: (1) novel junctions specific to the proband were present with ≥20 supporting reads, (2) canonical junctions present in parents were absent or reduced >5-fold in the proband (≤2 reads), and (3) the aberrant splicing pattern was consistent with the predicted effect (splice donor/acceptor loss).

**Mono-allelic expression analysis**

To identify regions of mono-allelic expression (MAE), we integrated DNA sequencing data with RNA sequencing for the 10 trios with available transcriptomic data. First, heterozygous SNPs were identified from WGS data where the proband was heterozygous and parental origin could be determined. RNA sequencing data was then analysed using GATK’s ASEReadCounter to quantify allele-specific expression at these informative sites where coverage was ≥10 reads. Sites were classified as showing mono-allelic expression when >95% of reads originated from a single parental allele. The overall MAE rate was calculated as the proportion of testable sites showing mono-allelic expression.

**DNA methylation analysis**

DNA methylation profiling was performed using the Illumina Infinium MethylationEPIC BeadChip array on all 14 trios and one duo. Quality control for the methylation data was performed according to a cross-package Bioconductor workflow for analysing methylation array data (29). Probes were removed if they: (1) had detection p-values >0.01 in >10% of samples, (2) had <3 beads in at least 5% of samples, (3) were known cross-reactive probes, (4) contained SNPs at the CpG interrogation site, or (5) were located on sex chromosomes. All samples passed quality control filtering except one proband, which was excluded from downstream analysis. Beta values were normalised using functional normalisation to reduce technical variation while preserving biological differences.

**Epigenetic age acceleration analysis**

Tissue type and age both influence methylation patterns, making clock selection a critical methodological step (30). Epigenetic age was calculated using the Hannum blood-specific clock, which is optimized for blood samples, and the Horvath pan-tissue clock, which can be used for both children and adults. Age acceleration was defined as the difference between predicted epigenetic age and chronological age. Samples were grouped by disease status (CHD-only, NDD-only, control) and by family relationship (proband, mother, father). To account for family structure, linear mixed-effects models were fitted with family as a random effect, using age and sex as covariates. Group differences in age acceleration were assessed using ANOVA and pairwise t-tests between disease groups.

## **Cross-cohort differential DNA methylation analysis**

Differential DNA methylation analysis was performed by integrating publicly available reference cohorts with the study cohort to enable individual patient-level comparisons. Two reference control datasets were obtained from the Gene Expression Omnibus: GSE74432 (n=53 controls, Illumina 450K array, mean age=10.8±4.8 years)(31) and GSE97362 (n=125 controls, Illumina 450K array, mean age=11.4±5.2 years)(32). Both datasets were from whole blood samples. These were combined with the study samples (n=15 probands, Illumina EPIC array) to create the complete study cohort. All samples passed quality control filtering except the proband previously mentioned, which was excluded.

Raw IDAT files from each platform were processed using the minfi package (v1.46.0) in R (v4.3.0). Cross-platform normalization was performed using preprocessNoob with dye bias correction to account for technical variation between 450K and EPIC arrays. Only probes that passed QC and were present in all three cohorts were included in downstream analysis. Beta values were converted to M-values using the logit transformation for statistical analysis. Batch effects between datasets and array platforms were corrected using ComBat from the sva package (v3.48.0), with negative control probe intensities included as covariates to preserve biological signal while removing technical variation. The effectiveness of batch correction was evaluated using principal component analysis on the 5,000 most variable probes, which showed batch effects had been removed (Supplementary Figure 1). Cell type composition was estimated using the EpiDISH package (v2.16.0) with the IDOL reference panel optimised for whole blood, yielding estimates for B cells, NK cells, CD4+ T cells, CD8+ T cells, monocytes, neutrophils, and eosinophils. A second analysis of epigenetic age was performed to compare the study probands against healthy controls. Two clocks, the EN clock and Levine clock, were used as no other clocks had sufficient CpG coverage. Epigenetic ages were compared between disease groups (CHD-only, NDD-only, controls) using ANOVA, with pairwise comparisons performed using Tukey’s HSD test.

Individual proband methylation profiles were compared to the control cohort. For each proband, M-values from the patient and control samples were subset, and a linear model was fitted using limma (v3.56.2) with a design matrix incorporating group status (patient vs. control), batch, and estimated cell type proportions as covariates. Contrasts were used to compare each patient to controls, and empirical Bayes moderation was applied with robust variance estimation. Differentially methylated probes (DMPs) were identified at FDR < 0.05 (Benjamini-Hochberg correction). This analysis was performed separately for each proband; patients with >10,000 significant DMPs were considered to have methylation dysregulation.

**Methylation pathway analysis**

Separately from the cross-cohort differential probe analysis described above, a within-cohort regional methylation analysis was performed to identify differentially methylated regions (DMRs) among study participants only. This analysis did not incorporate the external reference cohorts and was restricted to the study samples.

DMRs were identified using the MethylMiner pipeline (33), which detects statistically significant outlier DMR events within a defined cohort. Regions were filtered to those containing ≥ 5 probes within a 500bp window to ensure robustness. Genes annotated to proband-specific DMRs were subsequently assessed for pathway enrichment using clusterProfiler (34) with Gene Ontology Biological Process terms (35, 36).

**Supplementary Figure 1. Assessment of batch correction for cross-cohort DNA methylation data using principal component analysis (PCA).** Principal component analysis was performed on the 5,000 most variable probes prior to and following batch correction. Top panels show samples coloured by dataset (GSE74432, GSE97362, and the current study), and bottom panels show samples coloured by clinical group (CHD proband, NDD proband, and control). Before batch correction (left panels), samples cluster predominantly by dataset, indicating strong batch effects. After correction using ComBat (right panels), samples no longer segregate by dataset, demonstrating effective removal of technical variation while preserving biological heterogeneity across clinical groups. Percent variance explained by each principal component is indicated on the axes.

**Supplementary Figure 2. Polygenic risk scores for common neurodevelopmental and psychiatric phenotypes in patients with NDD and/or CHD compared with healthy control subjects.** Polygenic risk scores (PRS) were calculated for attention deficit hyperactivity disorder (ADHD), autism spectrum disorder (ASD), depression, and anxiety in patients with isolated congenital heart disease (CHD, n=1), isolated neurodevelopmental disorder (NDD, n=7), and comorbid CHD+NDD (n=1). Scores were standardised to a healthy control population (n=3,823). Data points represent the mean percentage difference compared to controls, with error bars indicating 95% confidence intervals.

**A.**

**B.**

**Supplementary Figure 3. RNA sequencing validation of splice-site variants.** Sashimi plots showing read coverage and splice junction usage at each variant locus. (A) *ARID1B* (NM_001374828.1:c.4479G>A) in Proband 19. The *de novo* variant disrupts the splice donor site, resulting in exon skipping. This aberrant splicing pattern is present in the proband but absent in the parents. (B) *COL18A1* (NM_001379500.1:c.3495+2T>C) in Proband 15. The paternally inherited variant disrupts the splice donor site, causing aberrant splicing in the proband and the father but not the mother. Junction read arcs are shown above the coverage tracks; arc thickness is proportional to the number of supporting reads.

**Supplementary Figure 4.** **Epigenetic age estimates by disease group using EN and Levine clocks.** Mean epigenetic ages are shown for CHD probands (n=3), controls (n=178), and NDD probands (n=11). Red bars represent EN clock estimates, and blue bars represent Levine clock estimates. Error bars indicate standard error of the mean.

**Supplementary Table 1. Data types available for each study participant.**

| Patient | Whole genome sequencing | RNA sequencing | DNA methylation |
| --- | --- | --- | --- |
| 1.001 | X | X | X |
| 1.002 | X | X | X |
| 1.003 | X | X | X |
| 2.001 | X | X | X |
| 2.002 | X | X | X |
| 2.003 | X | X | X |
| 3.001 | X | X | X |
| 3.002 | X | X | X |
| 3.003 | X | X | X |
| 4.001 | X |  | X |
| 4.002 | X |  | X |
| 4.003 | X |  | X |
| 5.001 | X |  | X |
| 5.002 | X |  | X |
| 5.003 | X |  | X |
| 6.001 | X |  | X |
| 6.002 | X |  | X |
| 6.003 | X |  | X |
| 8.001 | X | X | X |
| 8.002 | X | X | X |
| 9.001 | X | X | X |
| 9.002 | X | X | X |
| 9.003 | X | X | X |
| 11.001 |  |  | X |
| 11.002 |  |  | X |
| 12.001 | X | X | X |
| 12.002 | X | X | X |
| 12.003 | X | X | X |
| 13.001 | X | X | X |
| 13.002 | X | X | X |
| 13.003 | X | X | X |
| 15.001 | X | X | X |
| 15.002 | X | X | X |
| 15.003 | X | X | X |
| 16.001 | X |  | X |
| 16.002 | X |  | X |
| 16.003 | X |  | X |
| 19.001 | X | X | X |
| 19.002 | X | X | X |
| 19.003 | X | X | X |
| 20.001 | X | X | X |
| 20.002 | X | X | X |
| 20.003 | X | X | X |

**Supplementary Table 2. Neurodevelopmental disorder (NDD) and congenital heart disease (CHD) genes used for variant curation**. The gene list comprises NDD genes from Sanchis-Juan, Megy (1) and Blue, Ip (2), plus CHD genes from the CHD Gene database (<https://chdgene.victorchang.edu.au/>) and emerging CHD genes collected in-house. The Sanchis-Juan et al. list was derived from OMIM, DDG2P, PanelApp, and literature searches.

| A1CF | DNM2 | LRPPRC | ROBO4 |
| --- | --- | --- | --- |
| AAAS | DNMT1 | LRRC6 | ROCK1 |
| AARS | DNMT3A | LRRK2 | ROCK2 |
| AARS1 | DNMT3B | LTBP2 | ROGDI |
| AARS2 | DOCK6 | LTBP3 | ROR2 |
| AASS | DOCK8 | LTBP4 | RORA |
| ABAT | DOHH | LYST | RORB |
| ABCA2 | DOK7 | LZTR1 | RPE65 |
| ABCB11 | DOLK | MAB21L2 | RPGR |
| ABCB7 | DPAGT1 | MACF1 | RPGRIP1 |
| ABCC6 | DPF2 | MAF | RPGRIP1L |
| ABCC9 | DPH1 | MAFB | RPL10 |
| ABCD1 | DPM1 | MAGEL2 | RPL11 |
| ABCD4 | DRD2 | MAGI1 | RPL15 |
| ABHD12 | DRD5 | MAML2 | RPL18 |
| ABHD5 | DSPP | MAN1B1 | RPL26 |
| ABI1 | DST | MAN2B1 | RPL27 |
| ABLIM1 | DSTYK | MANBA | RPL35 |
| ACAD9 | DTNA | MAOA | RPL5 |
| ACADM | DVL1 | MAP2 | RPS17 |
| ACADS | DVL3 | MAP2K1 | RPS19 |
| ACADVL | DYM | MAP2K2 | RPS24 |
| ACAN | DYNC1H1 | MAP3K1 | RPS26 |
| ACAT1 | DYNC2H1 | MAP3K7 | RPS27 |
| ACO2 | DYNC2I1 | MAP3K7CL | RPS28 |
| ACOX1 | DYNC2I2 | MAPK1 | RPS29 |
| ACP5 | DYNC2LI1 | MAPK8IP3 | RPS6KA3 |
| ACP6 | DYRK1A | MAPKAPK5 | RPSA |
| ACSL4 | E2F4 | MAPRE1 | RRM2B |
| ACTA1 | EBF3 | MAPRE2 | RSPH1 |
| ACTA2 | EBP | MAPT | RSPH3 |
| ACTB | ECE1 | MARS2 | RSPH4A |
| ACTG1 | ECE2 | MASP1 | RSPH9 |
| ACTR1A | ECEL1 | MATN3 | RSPO4 |
| ACVRL1 | ECT2L | MATR3 | RTEL1 |
| ACY1 | EDA | MBD5 | RTN2 |
| ADA | EDNRA | MBD6 | RTN4IP1 |
| ADAM17 | EDNRB | MC2R | RTTN |
| ADAM19 | EED | MCCC1 | RUNX2 |
| ADAMTS19 | EEF1A2 | MCCC2 | RXYLT1 |
| ADAMTS6 | EFHC1 | MCEE | RYR1 |
| ADAMTSL4 | EFNB1 | MCOLN1 | RYR2 |
| ADAR | EFNB2 | MCPH1 | RYR3 |
| ADCY5 | EFTUD2 | MCTP2 | SACS |
| ADD3 | EGFR | MDH2 | SALL1 |
| ADGRG1 | EGR2 | MDM4 | SALL4 |
| ADGRG6 | EHMT1 | MEA1 | SAMD12 |
| ADGRV1 | EIF2AK3 | MECP2 | SAMD4A |
| ADNP | EIF4A2 | MECR | SAMHD1 |
| ADSL | EIF4A3 | MED12 | SATB2 |
| AFF2 | EIF6 | MED13 | SBDS |
| AFF4 | ELAC2 | MED13L | SBF1 |
| AFG3L2 | ELN | MED17 | SBF2 |
| AGA | ELOVL4 | MEF2C | SC5D |
| AGK | ELOVL5 | MEGF10 | SCAF8 |
| AGL | ENAH | MEGF8 | SCAMP2 |
| AGO1 | ENPP1 | MEIS1 | SCAPER |
| AGPS | EOGT | MEIS2 | SCARB2 |
| AGXT | EP300 | MESDC2 | SCARF2 |
| AHDC1 | EPG5 | MESP2 | SCN11A |
| AHI1 | EPHB4 | MFRP | SCN1A |
| AIFM1 | EPM2A | MFSD2A | SCN1B |
| AIMP1 | EPRS | MFSD8 | SCN2A |
| AIPL1 | EPRS1 | MGAT2 | SCN4A |
| AIRE | EPS15 | MGP | SCN5A |
| AK2 | ERBB3 | MIB1 | SCN8A |
| AKAP6 | ERBB4 | MICU1 | SCN9A |
| AKR1D1 | ERCC1 | MID1 | SCO1 |
| AKT1 | ERCC2 | MINK1 | SCO2 |
| AKT3 | ERCC3 | MIPEP | SDCCAG8 |
| ALAD | ERCC4 | MITF | SDHA |
| ALDH18A1 | ERCC5 | MKKS | SDHAF1 |
| ALDH1A2 | ERCC6 | MKS1 | SEC23B |
| ALDH1A3 | ERCC6L2 | MLC1 | SEMA3A |
| ALDH3A2 | ERCC8 | MLYCD | SEMA3D |
| ALDH4A1 | ERF | MMAA | SEMA3E |
| ALDH5A1 | ERLIN2 | MMAB | SEPSECS |
| ALDH7A1 | ESCO2 | MMACHC | SESN1 |
| ALDOA | ETFA | MMADHC | SET |
| ALDOB | ETFB | MMP13 | SETBP1 |
| ALG1 | ETFDH | MMP15 | SETD2 |
| ALG12 | ETHE1 | MMP21 | SETD5 |
| ALG13 | ETS1 | MMUT | SETX |
| ALG3 | EVC | MNX1 | SF3B4 |
| ALG6 | EVC2 | MOCS1 | SGCE |
| ALG8 | EVT2 | MOCS2 | SGPL1 |
| ALMS1 | EXOSC3 | MPDU1 | SGSH |
| ALPL | EXT1 | MPI | SH3PXD2B |
| ALS2 | EXT2 | MPLKIP | SH3TC2 |
| ALX1 | EXTL3 | MPV17 | SHANK2 |
| ALX3 | EYA1 | MPZ | SHANK3 |
| ALX4 | EYA3 | MRAS | SHH |
| AMBRA1 | EYS | MRE11 | SHOC2 |
| AMER1 | EZH1 | MRPS22 | SHOX |
| AMOTL1 | EZH2 | MRPS34 | SHOX2 |
| AMPD2 | FA2H | MSL3 | SHROOM3 |
| AMT | FAH | MSMO1 | SIGMAR1 |
| ANK3 | FAM111A | MSX1 | SIK1 |
| ANKH | FAM111B | MSX2 | SIL1 |
| ANKHD1 | FAM126A | MT-ATP6 | SIM1 |
| ANKRD1 | FAM135A | MT-ND1 | SIN3A |
| ANKRD11 | FAM160A2 | MT-ND4 | SIPA1L2 |
| ANO10 | FAM161A | MT-RNR1 | SIX1 |
| ANO3 | FAM181B | MT-TK | SIX3 |
| ANO5 | FAM20A | MT-TL1 | SIX5 |
| ANTXR1 | FAM20C | MT-TP | SKI |
| AP1G1 | FAM58A | MTHFR | SKIV2L |
| AP1S2 | FANCA | MTM1 | SLC12A2 |
| AP4B1 | FANCB | MTMR2 | SLC12A5 |
| AP4E1 | FANCC | MTMR3 | SLC12A6 |
| AP4M1 | FANCD2 | MTMR4 | SLC13A5 |
| AP4S1 | FANCE | MTO1 | SLC16A2 |
| AP5Z1 | FANCF | MTOR | SLC17A5 |
| APBB1 | FANCG | MTPAP | SLC19A3 |
| APBB3 | FANCI | MTR | SLC20A2 |
| APC | FANCL | MTRFR | SLC22A5 |
| APOB | FAR1 | MTRR | SLC25A15 |
| APOPT1 | FARS2 | MTTP | SLC25A20 |
| APTX | FARSB | MUT | SLC25A22 |
| AR | FAT1 | MYBPC1 | SLC25A26 |
| ARFGEF2 | FAT4 | MYCN | SLC25A38 |
| ARG1 | FBLN1 | MYH3 | SLC26A2 |
| ARHGAP29 | FBLN2 | MYH6 | SLC27A4 |
| ARHGAP31 | FBN1 | MYH8 | SLC2A1 |
| ARHGEF11 | FBN2 | MYH9 | SLC2A10 |
| ARHGEF12 | FBP1 | MYO15A | SLC2A2 |
| ARHGEF9 | FBRSL1 | MYO16 | SLC33A1 |
| ARID1A | FBXL4 | MYO18A | SLC35A1 |
| ARID1B | FBXO11 | MYO5A | SLC35A2 |
| ARID2 | FBXO7 | MYO5B | SLC35C1 |
| ARID3B | FEZ1 | MYO7A | SLC35D1 |
| ARL6 | FGD1 | MYOCD | SLC37A4 |
| ARMC4 | FGD4 | MYOM2 | SLC39A13 |
| ARMC9 | FGD5 | MYRF | SLC39A14 |
| ARSA | FGF10 | MYT1L | SLC39A8 |
| ARSB | FGF12 | NAA10 | SLC46A1 |
| ARSE | FGF14 | NAA15 | SLC4A1 |
| ARSL | FGF3 | NACC1 | SLC4A11 |
| ARV1 | FGF8 | NADSYN1 | SLC4A2 |
| ARX | FGFR1 | NAGA | SLC4A4 |
| ASAH1 | FGFR2 | NAGLU | SLC52A2 |
| ASH1L | FGFR3 | NAGS | SLC52A3 |
| ASL | FH | NALCN | SLC5A5 |
| ASNS | FHL1 | NANS | SLC6A1 |
| ASPA | FHOD3 | NBAS | SLC6A17 |
| ASPM | FIG4 | NBEA | SLC6A19 |
| ASS1 | FKBP14 | NBN | SLC6A3 |
| ASXL1 | FKRP | NCKAP1 | SLC6A5 |
| ASXL3 | FKTN | ND1 | SLC6A8 |
| ATAD1 | FLAD1 | ND4 | SLC9A6 |
| ATAD3A | FLNA | NDE1 | SLIT2 |
| ATCAY | FLNB | NDP | SLIT3 |
| ATE1 | FLNC | NDRG1 | SLX4 |
| ATG2A | FLT4 | NDUFA1 | SMAD1 |
| ATIC | FLVCR1 | NDUFA9 | SMAD3 |
| ATL1 | FLVCR2 | NDUFAF6 | SMAD4 |
| ATL2 | FMR1 | NDUFS1 | SMAD5 |
| ATM | FOLR1 | NDUFS4 | SMARCA2 |
| ATN1 | FOXC1 | NDUFS7 | SMARCA4 |
| ATP13A1 | FOXC2 | NDUFS8 | SMARCAL1 |
| ATP13A2 | FOXE1 | NDUFV1 | SMARCB1 |
| ATP1A3 | FOXE3 | NECTIN1 | SMARCC1 |
| ATP2B1 | FOXF1 | NECTIN4 | SMARCC2 |
| ATP2B4 | FOXG1 | NEFH | SMARCE1 |
| ATP2C1 | FOXL1 | NEFL | SMC1A |
| ATP6 | FOXN1 | NEK1 | SMC3 |
| ATP6AP2 | FOXO1 | NEK2 | SMCHD1 |
| ATP6V1B1 | FOXP1 | NEK8 | SMG7 |
| ATP6V1B2 | FOXP2 | NEU1 | SMO |
| ATP7A | FOXP3 | NEXMIF | SMOC1 |
| ATP7B | FOXP4 | NF1 | SMPD1 |
| ATP8B1 | FOXRED1 | NFATC1 | SMPD4 |
| ATP8B2 | FRA10AC1 | NFATC4 | SMPX |
| ATR | FRAS1 | NFE2L2 | SMS |
| ATRX | FREM1 | NFIA | SMURF1 |
| ATXN1 | FREM2 | NFIX | SNAP25 |
| ATXN10 | FRMD7 | NFU1 | SNCA |
| ATXN2 | FRYL | NGLY1 | SNORD118 |
| ATXN3 | FRZB | NHEJ1 | SNRPB |
| ATXN7 | FTCD | NHLRC1 | SNX14 |
| AUH | FTL | NHS | SON |
| AUTS2 | FTSJ1 | NIPA1 | SOS1 |
| B3GALNT2 | FUCA1 | NIPBL | SOS2 |
| B3GALT6 | FXN | NKX2-1 | SOX10 |
| B3GALTL | FYCO1 | NKX2-5 | SOX11 |
| B3GLCT | FZD10 | NKX3-2 | SOX13 |
| B4GALNT1 | FZD2 | NLRP3 | SOX17 |
| B4GALT7 | FZD5 | NMNAT1 | SOX18 |
| BBS1 | FZD6 | NNT | SOX2 |
| BBS10 | G6PC3 | NODAL | SOX3 |
| BBS12 | GAA | NOG | SOX5 |
| BBS2 | GABRA1 | NONO | SOX7 |
| BBS4 | GABRA2 | NOP2 | SOX9 |
| BBS5 | GABRB3 | NOP56 | SPAG1 |
| BBS7 | GABRG2 | NOTCH2 | SPART |
| BBS9 | GAD1 | NOTCH2NL | SPAST |
| BCAP31 | GALC | NOVA2 | SPATA5 |
| BCAR1 | GALE | NOX2 | SPEG |
| BCKDHA | GALK1 | NPC1 | SPEN |
| BCKDHB | GALNS | NPC2 | SPG11 |
| BCKDK | GALNT11 | NPHP1 | SPG21 |
| BCL11A | GALT | NPHP3 | SPG7 |
| BCL11B | GAMT | NPHP4 | SPINK5 |
| BCL9 | GANAB | NPHS1 | SPR |
| BCL9L | GARS | NPHS2 | SPRED1 |
| BCOR | GARS1 | NPR2 | SPRED2 |
| BCS1L | GAS8 | NPRL3 | SPTAN1 |
| BEAN1 | GATA2 | NR2F1 | SPTB |
| BFSP2 | GATA4 | NR2F2 | SPTBN1 |
| BGN | GATA6 | NR5A1 | SPTBN2 |
| BHLHA9 | GATAD2B | NRAS | SPTBN4 |
| BICC1 | GATM | NRK | SPTBN5 |
| BICD2 | GBA | NRP1 | SPTLC2 |
| BIN1 | GBA2 | NRXN1 | SRCAP |
| BLM | GCC2 | NRXN3 | SRD5A3 |
| BMP1 | GCDH | NSD1 | SRRM2 |
| BMP2 | GCH1 | NSD2 | SRY |
| BMP4 | GDAP1 | NSDHL | SSH1 |
| BMPER | GDF5 | NSUN2 | SSH2 |
| BMPR1A | GDF6 | NT5C2 | ST3GAL3 |
| BMPR1B | GDI1 | NT5C3A | ST3GAL5 |
| BOD1L1 | GET1 | NTRK1 | STAG1 |
| BOLA3 | GFAP | NTRK2 | STAG2 |
| BPNT2 | GFER | NTRK3 | STAMBP |
| BPTF | GFM1 | NUB1 | STAR |
| BRAF | GHR | NUBPL | STAT1 |
| BRAT1 | GJA1 | NUP107 | STAT6 |
| BRCA1 | GJA3 | NUS1 | STIL |
| BRCA2 | GJA5 | NYX | STK38 |
| BRD4 | GJA8 | OBSL1 | STK4 |
| BRF1 | GJB1 | OCRL | STRA6 |
| BRIP1 | GJB2 | ODAD1 | STRADA |
| BRK1 | GJB3 | ODAD2 | STS |
| BRPF1 | GJC1 | ODAPH | STUB1 |
| BRWD1 | GJC2 | OFD1 | STX1B |
| BRWD3 | GK | OGT | STXBP1 |
| BSCL2 | GLB1 | OPA3 | SUCLA2 |
| BSND | GLDC | OPHN1 | SUCLG1 |
| BTBD1 | GLE1 | OR52M1 | SULF1 |
| BTD | GLI1 | ORC1 | SUMF1 |
| BTK | GLI2 | ORC4 | SUPT3H |
| BUB1B | GLI3 | ORC6 | SUPT5H |
| C10orf2 | GLIS3 | OSGEP | SURF1 |
| C12orf57 | GLMN | OSTC | SUV420H1 |
| C12orf65 | GLUD1 | OTC | SUZ12 |
| C19orf12 | GLUL | OTOGL | SYNE1 |
| C1orf106 | GLYR1 | OTUD5 | SYNE2 |
| C2orf71 | GM2A | OTUD6B | SYNGAP1 |
| C4orf26 | GMPPA | OTULIN | SYNJ1 |
| C5orf42 | GMPPB | OTX2 | SYP |
| C8orf37 | GNA12 | OXCT1 | SZT2 |
| C9orf72 | GNAI1 | P3H1 | TAB2 |
| CA2 | GNAI3 | PACS1 | TAF1 |
| CA8 | GNAL | PACS2 | TAF6 |
| CABIN1 | GNAO1 | PAFAH1B1 | TAFAZZIN |
| CACNA1A | GNAS | PAH | TANGO2 |
| CACNA1C | GNB1 | PAK3 | TARDBP |
| CACNA1D | GNPAT | PALB2 | TAT |
| CACNA1E | GNPTAB | PAN2 | TAZ |
| CACNA1G | GNPTG | PANK2 | TBC1D23 |
| CACNA1H | GNS | PAPSS2 | TBC1D24 |
| CACNA1S | GORAB | PARK2 | TBCD |
| CACNB4 | GOSR2 | PARK7 | TBCE |
| CAD | GOT2 | PARN | TBCK |
| CAMK2A | GPC3 | PAX1 | TBL1XR1 |
| CAMK2B | GPC4 | PAX2 | TBP |
| CAMTA1 | GPC5 | PAX3 | TBX1 |
| CANX | GPC6 | PAX6 | TBX15 |
| CASK | GPR179 | PAX8 | TBX18 |
| CASQ1 | GPR98 | PAX9 | TBX2 |
| CASR | GPSM2 | PC | TBX20 |
| CASZ1 | GPT2 | PCARE | TBX22 |
| CATSPERG | GRB10 | PCBD1 | TBX3 |
| CBL | GRHL3 | PCCA | TBX4 |
| CBS | GRIA3 | PCCB | TBX5 |
| CBX5 | GRIA4 | PCDH19 | TBXAS1 |
| CC2D1A | GRID2 | PCGF2 | TCERG1 |
| CC2D2A | GRIK2 | PCK2 | TCF12 |
| CCBE1 | GRIN1 | PCM1 | TCF20 |
| CCDC103 | GRIN2A | PCNT | TCF4 |
| CCDC11 | GRIN2B | PCSK5 | TCF7L1 |
| CCDC114 | GRK1 | PCSK6 | TCF7L2 |
| CCDC115 | GRM1 | PCYT1A | TCN2 |
| CCDC22 | GRM6 | PDCD10 | TCOF1 |
| CCDC39 | GRN | PDE10A | TCTN2 |
| CCDC40 | GTF2H5 | PDE4D | TCTN3 |
| CCDC65 | GTPBP2 | PDE6B | TDGF1 |
| CCDC88C | GTPBP3 | PDE6G | TECPR2 |
| CCN1 | GUCY2C | PDGFB | TEK |
| CCND2 | GUSB | PDGFRA | TENM3 |
| CCNO | H1-4 | PDGFRB | TENM4 |
| CCNQ | HACE1 | PDHA1 | TEP1 |
| CCT5 | HADH | PDHX | TERT |
| CD80 | HADHA | PDSS2 | TET3 |
| CDC45 | HAS2 | PDYN | TFAP2A |
| CDC6 | HAX1 | PEPD | TFAP2B |
| CDH15 | HCCS | PEX1 | TFG |
| CDH2 | HCFC1 | PEX10 | TGDS |
| CDH23 | HCN1 | PEX11B | TGFB1 |
| CDH3 | HCN2 | PEX12 | TGFB2 |
| CDH8 | HDAC2 | PEX13 | TGFB3 |
| CDK13 | HDAC4 | PEX14 | TGFBR1 |
| CDK5RAP2 | HDAC7 | PEX16 | TGFBR2 |
| CDKL5 | HDAC8 | PEX19 | TGIF1 |
| CDKN1C | HDAC9 | PEX2 | TGM6 |
| CDON | HECW2 | PEX26 | TH |
| CDT1 | HERC1 | PEX3 | THAP1 |
| CELSR1 | HERC3 | PEX5 | THOC6 |
| CEMIP2 | HEXA | PEX6 | THRA |
| CENPJ | HEXB | PEX7 | TIAM1 |
| CEP104 | HEY1 | PGAP1 | TIMM50 |
| CEP152 | HEY2 | PGAP2 | TIMM8A |
| CEP290 | HGSNAT | PGAP3 | TINF2 |
| CEP41 | HIBCH | PGK1 | TJP2 |
| CEP57 | HINT1 | PGM1 | TK2 |
| CEP63 | HIST1H1E | PGM3 | TKT |
| CEP83 | HIVEP2 | PHF6 | TLK2 |
| CERT1 | HK1 | PHF8 | TLN1 |
| CFAP298 | HK2 | PHGDH | TMCO1 |
| CFAP300 | HLCS | PHIP | TMED8 |
| CFAP418 | HMBS | PHKA1 | TMEM126B |
| CFAP54 | HMCN1 | PHOX2B | TMEM165 |
| CFAP74 | HMGA2 | PIAS1 | TMEM216 |
| CFH | HMGCL | PIAS3 | TMEM237 |
| CHAMP1 | HMGCS2 | PIEZO2 | TMEM240 |
| CHD2 | HNF1B | PIGA | TMEM5 |
| CHD4 | HNF4A | PIGG | TMEM67 |
| CHD7 | HNRNPAB | PIGL | TMEM70 |
| CHD8 | HNRNPC | PIGN | TMEM87B |
| CHL1 | HNRNPH1 | PIGO | TMEM94 |
| CHM | HNRNPH2 | PIGT | TMLHE |
| CHRDL1 | HNRNPK | PIGV | TMOD3 |
| CHRNA2 | HNRNPU | PIK3C2B | TMPRSS6 |
| CHRNA4 | HOXA1 | PIK3C2G | TMTC2 |
| CHRNB2 | HOXA13 | PIK3CA | TMTC3 |
| CHRNG | HOXC13 | PIK3R1 | TNPO1 |
| CHST14 | HOXD13 | PIK3R2 | TNR |
| CHST3 | HPD | PINK1 | TNS1 |
| CHSY1 | HPGD | PITRM1 | TOE1 |
| CHUK | HPRT1 | PITX2 | TOP3A |
| CIB2 | HPS1 | PITX3 | TOR1A |
| CIROP | HPSE2 | PKD1 | TP53RK |
| CISD2 | HR | PKD1L1 | TP63 |
| CKAP2L | HRAS | PKD2 | TPM1 |
| CLCN2 | HSD17B10 | PKHD1 | TPP1 |
| CLCN7 | HSD17B4 | PKHD1L1 | TPRKB |
| CLDN19 | HSD3B7 | PLA2G6 | TRAF7 |
| CLDN9 | HSF4 | PLAA | TRAP2 |
| CLN3 | HSP90AA1 | PLB1 | TRAPPC11 |
| CLN5 | HSPA2 | PLCB1 | TRAPPC2 |
| CLN6 | HSPA9 | PLCE1 | TRAPPC9 |
| CLN8 | HSPD1 | PLD1 | TRDN |
| CLP1 | HSPG2 | PLEC | TREX1 |
| CLPB | HTT | PLEKHO1 | TRIM32 |
| CLSPN | HUWE1 | PLK4 | TRIM37 |
| CLUH | HYAL1 | PLOD1 | TRIM71 |
| CNNM2 | HYAL2 | PLOD2 | TRIO |
| CNOT3 | HYDIN | PLP1 | TRIP11 |
| CNPY3 | HYLS1 | PLPBP | TRIP12 |
| CNTNAP1 | IDS | PLXNB1 | TRMT10A |
| CNTNAP2 | IDUA | PLXND1 | TRPM1 |
| COA8 | IER3IP1 | PMFBP1 | TRPM6 |
| COASY | IFIH1 | PMM2 | TRPS1 |
| COG1 | IFITM5 | PMP22 | TRPV4 |
| COG4 | IFT122 | PMPCB | TSC1 |
| COG7 | IFT140 | PMS2 | TSC2 |
| COG8 | IFT172 | PNKD | TSEN2 |
| COL10A1 | IFT43 | PNKP | TSEN54 |
| COL11A1 | IFT80 | PNPLA6 | TSHB |
| COL11A2 | IFT81 | PNPO | TSHR |
| COL18A1 | IGF1 | PNPT1 | TSPAN7 |
| COL1A1 | IGF1R | POC1A | TTBK2 |
| COL1A2 | IGF2 | POC1B | TTC19 |
| COL27A1 | IGHMBP2 | POGZ | TTC25 |
| COL2A1 | IGSF1 | POLD1 | TTC3 |
| COL3A1 | IHH | POLG | TTC37 |
| COL4A1 | IKBKG | POLR1C | TTC7A |
| COL4A2 | IL11RA | POLR1D | TTC8 |
| COL4A3 | IL1RAPL1 | POLR3A | TTLL10 |
| COL4A3BP | IMPAD1 | POLR3B | TTLL6 |
| COL4A4 | INO80 | POMGNT1 | TTPA |
| COL5A1 | INPP5E | POMGNT2 | TUBA1A |
| COL5A2 | INPP5K | POMT1 | TUBA8 |
| COL6A1 | INPPL1 | POMT2 | TUBB |
| COL6A2 | IPO8 | PORCN | TUBB2A |
| COL6A3 | IQGAP1 | POTEI | TUBB2B |
| COL9A1 | IQSEC2 | POU1F1 | TUBB3 |
| COL9A2 | IRF2BPL | PPA2 | TUBB4A |
| COL9A3 | IRF6 | PPEF2 | TUBGCP6 |
| COLEC11 | IRX1 | PPM1D | TUSC3 |
| COMP | IRX4 | PPP1CB | TWIST1 |
| COPS6 | ISL1 | PPP1R12A | TWIST2 |
| COQ2 | ISPD | PPP2CA | TWNK |
| COQ4 | ITGA1 | PPP2R1A | TXNDC3 |
| COQ8A | ITGA3 | PPP2R2B | TXNL4A |
| COQ9 | ITGA7 | PPP2R5D | TYK2 |
| COX10 | ITGB1 | PPT1 | TYR |
| COX15 | ITPA | PQBP1 | TYRP1 |
| COX6B1 | ITPR1 | PRDM1 | UBA5 |
| COX7B | ITSN1 | PRDM12 | UBAP2 |
| CP | IVD | PRDM5 | UBASH3B |
| CPA6 | JAG1 | PRICKLE1 | UBE2A |
| CPLANE1 | JAG2 | PRICKLE2 | UBE2D3 |
| CPS1 | JAGN1 | PRKACA | UBE3A |
| CPT2 | JAK3 | PRKAR1A | UBE3B |
| CRB1 | JAM3 | PRKCG | UBR1 |
| CRB2 | JARID2 | PRKD1 | UBR5 |
| CREBBP | JMJD6 | PRKD3 | UBR7 |
| CRKL | KANK2 | PRKN | UBTF |
| CRPPA | KANSL1 | PRKRA | UFC1 |
| CRX | KARS | PRMT5 | UFM1 |
| CRYAA | KARS1 | PRMT7 | UGDH |
| CRYBA1 | KAT6A | PROP1 | UGT1A1 |
| CRYBA4 | KAT6B | PROSC | UMPS |
| CRYBB1 | KBTBD13 | PRPS1 | UNC80 |
| CRYBB2 | KCNA1 | PRR12 | UPF3B |
| CRYBB3 | KCNA2 | PRRT2 | UROC1 |
| CRYGC | KCNB1 | PRSS12 | UROS |
| CRYGD | KCNC1 | PRSS56 | USB1 |
| CSF1R | KCNC3 | PRUNE1 | USH2A |
| CSNK2A1 | KCND3 | PRX | USP25 |
| CSPP1 | KCNE1 | PSAP | USP34 |
| CSRP1 | KCNH1 | PSEN1 | USP4 |
| CSTB | KCNH2 | PSMB8 | USP44 |
| CTC1 | KCNJ10 | PSMD12 | USP9X |
| CTCF | KCNJ11 | PSPH | UVSSA |
| CTDP1 | KCNMA1 | PTCH1 | VAMP1 |
| CTNNB1 | KCNQ1 | PTCHD1 | VAMP2 |
| CTNND1 | KCNQ2 | PTDSS1 | VARS2 |
| CTNS | KCNQ3 | PTEN | VDR |
| CTR9 | KCNQ5 | PTF1A | VEGFA |
| CTSA | KCNT1 | PTH1R | VIPAS39 |
| CTSD | KCNT2 | PTHLH | VLDLR |
| CTSF | KCTD1 | PTK2B | VPS13B |
| CTSK | KCTD10 | PTPDC1 | VPS13D |
| CUL4B | KCTD20 | PTPN11 | VPS33B |
| CUL7 | KCTD3 | PTPN23 | VPS35 |
| CUX1 | KCTD7 | PTPRB | VPS53 |
| CWC27 | KDM5A | PTPRF | VSX2 |
| CYB5R3 | KDM5B | PTS | WAC |
| CYC1 | KDM5C | PUF60 | WARS2 |
| CYFIP1 | KDM6A | PUM1 | WASF1 |
| CYP1B1 | KIAA0586 | PURA | WASF2 |
| CYP27A1 | KIAA1109 | PUS3 | WASHC5 |
| CYP2U1 | KIAA1715 | PVRL1 | WBP11 |
| CYP7B1 | KIAA2022 | PVRL4 | WDPCP |
| DAAM1 | KIDINS220 | PYCR1 | WDR11 |
| DAB1 | KIF11 | PYGL | WDR19 |
| DAG1 | KIF13A | PYGM | WDR26 |
| DAND5 | KIF14 | QARS | WDR27 |
| DAPK3 | KIF1A | QARS1 | WDR34 |
| DARS | KIF1BP | QDPR | WDR35 |
| DARS1 | KIF1C | QKI | WDR37 |
| DARS2 | KIF22 | QRICH1 | WDR4 |
| DBT | KIF5A | QSER1 | WDR45 |
| DCAF17 | KIF5C | RAB11B | WDR60 |
| DCC | KIF7 | RAB18 | WDR62 |
| DCHS1 | KIFBP | RAB23 | WDR70 |
| DCTN1 | KIRREL3 | RAB39B | WDR73 |
| DCX | KIT | RAB3GAP1 | WDR81 |
| DDB2 | KLF1 | RAB3GAP2 | WHSC1 |
| DDC | KLF13 | RABGAP1L | WHSC1L1 |
| DDHD1 | KLHL26 | RAC1 | WLS |
| DDHD2 | KLHL40 | RACGAP1 | WNK1 |
| DDOST | KMT2A | RAD21 | WNK4 |
| DDR2 | KMT2B | RAD54L2 | WNT1 |
| DDX11 | KMT2C | RAF1 | WNT10A |
| DDX3X | KMT2D | RAI1 | WNT10B |
| DEAF1 | KMT2E | RAI2 | WNT2 |
| DEPDC5 | KMT5B | RALGPS2 | WNT3 |
| DGKD | KNL1 | RANBP2 | WNT5A |
| DHCR24 | KPTN | RANBP9 | WNT7A |
| DHCR7 | KRAS | RAPSN | WRAP53 |
| DHFR | KRIT1 | RARB | WT1 |
| DHH | KTU | RARS2 | WWOX |
| DHODH | L1CAM | RASA1 | XPA |
| DHX30 | L2HGDH | RAX | XPC |
| DIAPH2 | LAGE3 | RBBP8 | XPNPEP2 |
| DIP2C | LAMA1 | RBM10 | XRCC4 |
| DIS3L2 | LAMA2 | RBM27 | XYLT1 |
| DISP1 | LAMA5 | RBM8A | YWHAG |
| DKC1 | LAMB1 | RBPMS | YY1 |
| DLAT | LAMC1 | RC3H2 | ZBTB18 |
| DLC1 | LAMC3 | RECQL4 | ZBTB20 |
| DLD | LAMP2 | REEP1 | ZC3H3 |
| DLG3 | LARGE1 | REEP2 | ZC4H2 |
| DLG4 | LARP1 | RELN | ZCCHC6 |
| DLL3 | LARP7 | RERE | ZDHHC20 |
| DLL4 | LBR | RET | ZDHHC9 |
| DMD | LDB3 | RETREG1 | ZEB2 |
| DMP1 | LEFTY2 | RFT1 | ZFAND5 |
| DMPK | LEMD3 | RFX3 | ZFP57 |
| DNAAF1 | LFNG | RFX6 | ZFYVE26 |
| DNAAF11 | LGI1 | RGS3 | ZIC1 |
| DNAAF3 | LGI4 | RHOBTB2 | ZIC2 |
| DNAAF4 | LHX3 | RIOK1 | ZIC3 |
| DNAH1 | LHX4 | RIPK4 | ZMIZ1 |
| DNAH10 | LIG4 | RIT1 | ZMIZ2 |
| DNAH12 | LIMCH1 | RLIM | ZMPSTE24 |
| DNAH2 | LIMS1 | RMND1 | ZMYM2 |
| DNAH3 | LINS | RMRP | ZMYM4 |
| DNAH6 | LINS1 | RNASEH2A | ZMYND11 |
| DNAH7 | LITAF | RNASEH2B | ZMYND8 |
| DNAH9 | LMBRD1 | RNASEH2C | ZNF148 |
| DNAI1 | LMNA | RNASET2 | ZNF292 |
| DNAI2 | LMX1B | RNF135 | ZNF407 |
| DNAJA4 | LNPK | RNF20 | ZNF462 |
| DNAJB6 | LOXHD1 | RNF216 | ZNF623 |
| DNAJC12 | LRP1 | RNF38 | ZNF699 |
| DNHD1 | LRP2 | RNU4ATAC | ZNF711 |
| DNM1 | LRP4 | ROBO1 | ZSWIM6 |
| DNM1L | LRP5 | ROBO3 |  |

**Supplementary Table 3. Aberrantly expressed genes identified by RNA sequencing in probands with neurodevelopmental disorders.**

| Gene | Sample ID | Adjusted P-value | zScore | l_2_fc |
| --- | --- | --- | --- | --- |
| *EEF1E1* | 3.001 | 0.042 | 5.3 | 1.49 |
| *U2AF1* | 3.001 | 0.042 | -4.63 | -4.4 |
| *RAMAC* | 3.001 | 0.042 | -5.27 | -0.79 |
| *RPL19* | 8.001 | 0.002 | -5.72 | -0.43 |
| *PPID* | 8.001 | 0.002 | -5.91 | -0.77 |
| *CDK5RAP2* | 9.001 | 0.036 | 4.18 | 0.58 |
| *EIF4G1* | 9.001 | 0.024 | -5.66 | -0.4 |
| *EIF2B5* | 9.001 | 0.001 | -6.85 | -0.59 |
| *DVL3* | 9.001 | 0.024 | -5.24 | -0.49 |
| *AP2M1* | 9.001 | 0.024 | -5.15 | -0.49 |
| *ABCF3* | 9.001 | 0.008 | -6.22 | -0.54 |
| *SENP2* | 9.001 | 0.009 | -5.6 | -0.54 |
| *PSMD2* | 9.001 | 0.009 | -5.91 | -0.49 |
| *PARL* | 9.001 | 0.011 | -5.86 | -0.7 |

**Supplementary Table 4. Top 25 Gene Ontology Biological Processes using differentially methylated regions.**

| GO Biological Process ID | Pathway Description | Adjusted P-value |
| --- | --- | --- |
| GO:0098742 | Cell-cell adhesion via plasma-membrane adhesion molecules | <0.0001 |
| GO:0007156 | Homophilic cell adhesion via plasma membrane adhesion molecules | <0.0001 |
| GO:0042391 | Regulation of membrane potential | 0.0001 |
| GO:0060537 | Muscle tissue development | 0.0001 |
| GO:0042692 | Muscle cell differentiation | 0.0001 |
| GO:0051146 | Striated muscle cell differentiation | 0.0002 |
| GO:0007389 | Pattern specification process | 0.0003 |
| GO:0030534 | Adult behaviour | 0.0003 |
| GO:0034329 | Cell junction assembly | 0.0003 |
| GO:0045165 | Cell fate commitment | 0.0003 |
| GO:1903522 | Regulation of blood circulation | 0.0003 |
| GO:0003002 | Regionalization | 0.0004 |
| GO:0007411 | Axon guidance | 0.0004 |
| GO:0097485 | Neuron projection guidance | 0.0004 |
| GO:0007517 | Muscle organ development | 0.0005 |
| GO:0002763 | Positive regulation of myeloid leukocyte differentiation | 0.0005 |
| GO:0048705 | Skeletal system morphogenesis | 0.0006 |
| GO:0048706 | Embryonic skeletal system development | 0.0007 |
| GO:0001763 | Morphogenesis of a branching structure | 0.0007 |
| GO:0048754 | Branching morphogenesis of an epithelial tube | 0.0008 |
| GO:0050808 | Synapse organization | 0.0008 |
| GO:0019226 | Transmission of nerve impulse | 0.0009 |
| GO:0008016 | Regulation of heart contraction | 0.0010 |
| GO:0048863 | Stem cell differentiation | 0.0012 |
| GO:0060078 | Regulation of postsynaptic membrane potential | 0.0015 |

**References**

1.	Sanchis-Juan A, Megy K, Stephens J, Armirola Ricaurte C, Dewhurst E, Low K, et al. Genome sequencing and comprehensive rare-variant analysis of 465 families with neurodevelopmental disorders. Am J Hum Genet. 2023;110(8):1343-55.

2.	Blue GM, Ip E, Walker K, Kirk EP, Loughran-Fowlds A, Sholler GF, et al. Genetic burden and associations with adverse neurodevelopment in neonates with congenital heart disease. Am Heart J. 2018;201:33-9.

3.	Zhang F, Flickinger M, Taliun SAG, In PPGC, Abecasis GR, Scott LJ, et al. Ancestry-agnostic estimation of DNA sample contamination from sequence reads. Genome Res. 2020;30(2):185-94.

4.	Purcell S, Neale B, Todd-Brown K, Thomas L, Ferreira MA, Bender D, et al. PLINK: a tool set for whole-genome association and population-based linkage analyses. Am J Hum Genet. 2007;81(3):559-75.

5.	Manichaikul A, Mychaleckyj JC, Rich SS, Daly K, Sale M, Chen WM. Robust relationship inference in genome-wide association studies. Bioinformatics. 2010;26(22):2867-73.

6.	McKenna A, Hanna M, Banks E, Sivachenko A, Cibulskis K, Kernytsky A, et al. The Genome Analysis Toolkit: a MapReduce framework for analyzing next-generation DNA sequencing data. Genome Res. 2010;20(9):1297-303.

7.	Li H, Durbin R. Fast and accurate long-read alignment with Burrows-Wheeler transform. Bioinformatics. 2010;26(5):589-95.

8.	Wang K, Li M, Hakonarson H. ANNOVAR: functional annotation of genetic variants from high-throughput sequencing data. Nucleic Acids Res. 2010;38(16):e164.

9.	McLaren W, Gil L, Hunt SE, Riat HS, Ritchie GR, Thormann A, et al. The Ensembl Variant Effect Predictor. Genome Biol. 2016;17(1):122.

10.	Karczewski KJ, Francioli LC, Tiao G, Cummings BB, Alfoldi J, Wang Q, et al. The mutational constraint spectrum quantified from variation in 141,456 humans. Nature. 2020;581(7809):434-43.

11.	Ioannidis NM, Rothstein JH, Pejaver V, Middha S, McDonnell SK, Baheti S, et al. REVEL: An Ensemble Method for Predicting the Pathogenicity of Rare Missense Variants. Am J Hum Genet. 2016;99(4):877-85.

12.	Feng BJ. PERCH: A Unified Framework for Disease Gene Prioritization. Hum Mutat. 2017;38(3):243-51.

13.	Robinson JT, Thorvaldsdottir H, Winckler W, Guttman M, Lander ES, Getz G, et al. Integrative genomics viewer. Nat Biotechnol. 2011;29(1):24-6.

14.	Kopanos C, Tsiolkas V, Kouris A, Chapple CE, Albarca Aguilera M, Meyer R, et al. VarSome: the human genomic variant search engine. Bioinformatics. 2019;35(11):1978-80.

15.	Pinese M, Lacaze P, Rath EM, Stone A, Brion MJ, Ameur A, et al. The Medical Genome Reference Bank contains whole genome and phenotype data of 2570 healthy elderly. Nat Commun. 2020;11(1):435.

16.	Lambert SA, Wingfield B, Gibson JT, Gil L, Ramachandran S, Yvon F, et al. Enhancing the Polygenic Score Catalog with tools for score calculation and ancestry normalization. Nat Genet. 2024;56(10):1989-94.

17.	Ewels PA, Peltzer A, Fillinger S, Patel H, Alneberg J, Wilm A, et al. The nf-core framework for community-curated bioinformatics pipelines. Nat Biotechnol. 2020;38(3):276-8.

18.	Di Tommaso P, Chatzou M, Floden EW, Barja PP, Palumbo E, Notredame C. Nextflow enables reproducible computational workflows. Nat Biotechnol. 2017;35(4):316-9.

19.	Chang CC, Chow CC, Tellier LC, Vattikuti S, Purcell SM, Lee JJ. Second-generation PLINK: rising to the challenge of larger and richer datasets. Gigascience. 2015;4:7.

20.	Zhang D, Dey R, Lee S. Fast and robust ancestry prediction using principal component analysis. Bioinformatics. 2020;36(11):3439-46.

21.	Gruning B, Dale R, Sjodin A, Chapman BA, Rowe J, Tomkins-Tinch CH, et al. Bioconda: sustainable and comprehensive software distribution for the life sciences. Nat Methods. 2018;15(7):475-6.

22.	Sato JR, Biazoli CE, Bueno APA, Caye A, Pan PM, Santoro M, et al. Polygenic risk score for attention-deficit/hyperactivity disorder and brain functional networks segregation in a community-based sample. Genes Brain Behav. 2023;22(2):e12838.

23.	Tanigawa Y, Qian J, Venkataraman G, Justesen JM, Li R, Tibshirani R, et al. Significant sparse polygenic risk scores across 813 traits in UK Biobank. PLoS Genet. 2022;18(3):e1010105.

24.	Grove J, Ripke S, Als TD, Mattheisen M, Walters RK, Won H, et al. Identification of common genetic risk variants for autism spectrum disorder. Nat Genet. 2019;51(3):431-44.

25.	Prive F, Aschard H, Carmi S, Folkersen L, Hoggart C, O'Reilly PF, et al. Portability of 245 polygenic scores when derived from the UK Biobank and applied to 9 ancestry groups from the same cohort. Am J Hum Genet. 2022;109(1):12-23.

26.	Dobin A, Davis CA, Schlesinger F, Drenkow J, Zaleski C, Jha S, et al. STAR: ultrafast universal RNA-seq aligner. Bioinformatics. 2013;29(1):15-21.

27.	Brechtmann F, Mertes C, Matuseviciute A, Yepez VA, Avsec Z, Herzog M, et al. OUTRIDER: A Statistical Method for Detecting Aberrantly Expressed Genes in RNA Sequencing Data. Am J Hum Genet. 2018;103(6):907-17.

28.	Deshwar AR, Yuki KE, Hou H, Liang Y, Khan T, Celik A, et al. Trio RNA sequencing in a cohort of medically complex children. Am J Hum Genet. 2023;110(5):895-900.

29.	Maksimovic J, Phipson B, Oshlack A. A cross-package Bioconductor workflow for analysing methylation array data. F1000Res. 2016;5:1281.

30.	Schuurmans IK, Felix JF, Suderman M, Yousefi P, Cecil CAM. Bringing methylation profile scores to early life. Nat Rev Genet. 2026;27(3):187-8.

31.	Choufani S, Cytrynbaum C, Chung BH, Turinsky AL, Grafodatskaya D, Chen YA, et al. NSD1 mutations generate a genome-wide DNA methylation signature. Nat Commun. 2015;6:10207.

32.	Butcher DT, Cytrynbaum C, Turinsky AL, Siu MT, Inbar-Feigenberg M, Mendoza-Londono R, et al. CHARGE and Kabuki Syndromes: Gene-Specific DNA Methylation Signatures Identify Epigenetic Mechanisms Linking These Clinically Overlapping Conditions. Am J Hum Genet. 2017;100(5):773-88.

33.	LaFlamme CW, Rastin C, Sengupta S, Pennington HE, Russ-Hall SJ, Schneider AL, et al. Diagnostic utility of DNA methylation analysis in genetically unsolved pediatric epilepsies and CHD2 episignature refinement. Nat Commun. 2024;15(1):6524.

34.	Yu G, Wang LG, Han Y, He QY. clusterProfiler: an R package for comparing biological themes among gene clusters. OMICS. 2012;16(5):284-7.

35.	Ashburner M, Ball CA, Blake JA, Botstein D, Butler H, Cherry JM, et al. Gene ontology: tool for the unification of biology. The Gene Ontology Consortium. Nat Genet. 2000;25(1):25-9.

36.	Gene Ontology C, Aleksander SA, Balhoff J, Carbon S, Cherry JM, Drabkin HJ, et al. The Gene Ontology knowledgebase in 2023. Genetics. 2023;224(1).
